# Application of polymersomes in membrane protein study and drug discovery: Progress, strategies, and perspectives

**DOI:** 10.1002/btm2.10350

**Published:** 2022-06-28

**Authors:** Chih Hung Lo, Jialiu Zeng

**Affiliations:** ^1^ Lee Kong Chian School of Medicine Nanyang Technological University Singapore Singapore; ^2^ Department of Neurology, Brigham and Women's Hospital, Harvard Medical School Boston Massachusetts USA; ^3^ Department of Biomedical Engineering Boston University Boston Massachusetts USA; ^4^ Department of Chemistry Boston University Boston Massachusetts USA

**Keywords:** biophysical characterization, drug discovery, high‐throughput screening, incorporation, liposome, nano‐vesicle, polymersome, proteoliposome, proteopolymersome, reconstitution

## Abstract

Membrane proteins (MPs) play key roles in cellular signaling pathways and are responsible for intercellular and intracellular interactions. Dysfunctional MPs are directly related to the pathogenesis of various diseases, and they have been exploited as one of the most sought‐after targets in the pharmaceutical industry. However, working with MPs is difficult given that their amphiphilic nature requires protection from biological membrane or membrane mimetics. Polymersomes are bilayered nano‐vesicles made of self‐assembled block copolymers that have been widely used as cell membrane mimetics for MP reconstitution and in engineering of artificial cells. This review highlights the prevailing trend in the application of polymersomes in MP study and drug discovery. We begin with a review on the techniques for synthesis and characterization of polymersomes as well as methods of MP insertion to form proteopolymersomes. Next, we review the structural and functional analysis of the different types of MPs reconstituted in polymersomes, including membrane transport proteins, MP complexes, and membrane receptors. We then summarize the factors affecting reconstitution efficiency and the quality of reconstituted MPs for structural and functional studies. Additionally, we discuss the potential in using proteopolymersomes as platforms for high‐throughput screening (HTS) in drug discovery to identify modulators of MPs. We conclude by providing future perspectives and recommendations on advancing the study of MPs and drug development using proteopolymersomes.

## 1 INTRODUCTION

Membrane proteins (MPs) constitute 20%–30% of all proteins encoded by the genome of various organisms^1^, ^2^, ^3^ and represent the targets of most pharmacological agents.^4^, ^5^, ^6^ MPs include signal transducers, channel proteins, metabolite transporters, cell surface receptors, enzymes, and anchors. Dysfunctional MPs are associated with various diseases including cancers, autoimmune diseases, and neurological disorders.^7^ Therefore, understanding both the structural and functional effects of MPs is of great importance. Currently, 6.5% of the over 181,969 entries of protein structures in the Protein Data Bank are MPs with structures deposited in different databases.^3^, ^8^, ^9^ Of these, only less than 2% have high‐resolution structures consistently found in all databases.^6^, ^10^ The dearth of studies that focus on MPs can be contributed by various factors. First, MPs are usually unstable and require a bilayer membrane for them to be folded correctly during protein translation. Second, it is difficult to obtain stable and functional MPs of interest in high yields, as MPs are usually low in numbers and tend to aggregate in the cytoplasm, despite attempts at protein overexpression.^6^, ^11^ Importantly, MPs are generally insoluble in aqueous solution due to the incompatibility between the hydrophobic nature of MP surfaces associated with lipid membranes and the hydrophilicity of solvent molecules. The use of amphiphilic agents is thus necessary to extract MPs from the native membranes and maintain them in a stable soluble form. Hence, there is a need to develop synthetic membrane platforms that mimic native biological membrane to provide amphiphilic environments for the MPs and maintain their structural and functional integrity for in vitro protein studies.^12^, ^13^, ^14^, ^15^

Conventional methods of MP study include the usage of protein tethered lipid bilayer and supported planar lipid bilayer membranes.^16^, ^17^ However, these systems have limitations such as incompatibility between tethered molecules and extra‐membranous domains, inaccessibility of region occupied by tethered molecules as well as uncontrollable orientation of inserted MPs and constraints on their biological functions.^16^, ^17^ Therefore, cell membrane mimetics with vesicular morphologies known as nano‐vesicles have been increasingly used to overcome these limitations.^18^ While liposomes are composed of natural nontoxic phospholipids, polymersomes are formed by amphiphilic block copolymers.^19^, ^20^ Both types of nano‐vesicles are analogous to biological membrane and suitable for MP residence.^17^, ^18^, ^20^, ^21^, ^22^ These nano‐vesicles, or small unilamellar vesicles (SUV), have a size of 20–100 nm and have the lowest interfacial area and highest configurational entropy as compared to other morphologies. This makes them more energetically favorable for MP reconstitution.^20^ They also have an increased stability over large unilamellar vesicles (LUVs, >100 nm in size) and giant unilamellar vesicles (GUVs, >1 μm in size).^20^ Additionally, they contain a concentration gradient, which can play a key role in determining the functions of pore‐forming channel MPs.^23^ While liposomes have been widely used and reviewed for their use in MP reconstitution and the related structural and functional studies,^17^ they are limited by low stability.^22^ To overcome this limitation, polymersomes have been increasingly adopted for MP studies because of their superior stability.^22^, ^23^, ^24^ Liposomes and polymersomes with reconstituted MPs are termed as proteoliposomes and proteopolymersomes, respectively.

**FIGURE 1:**
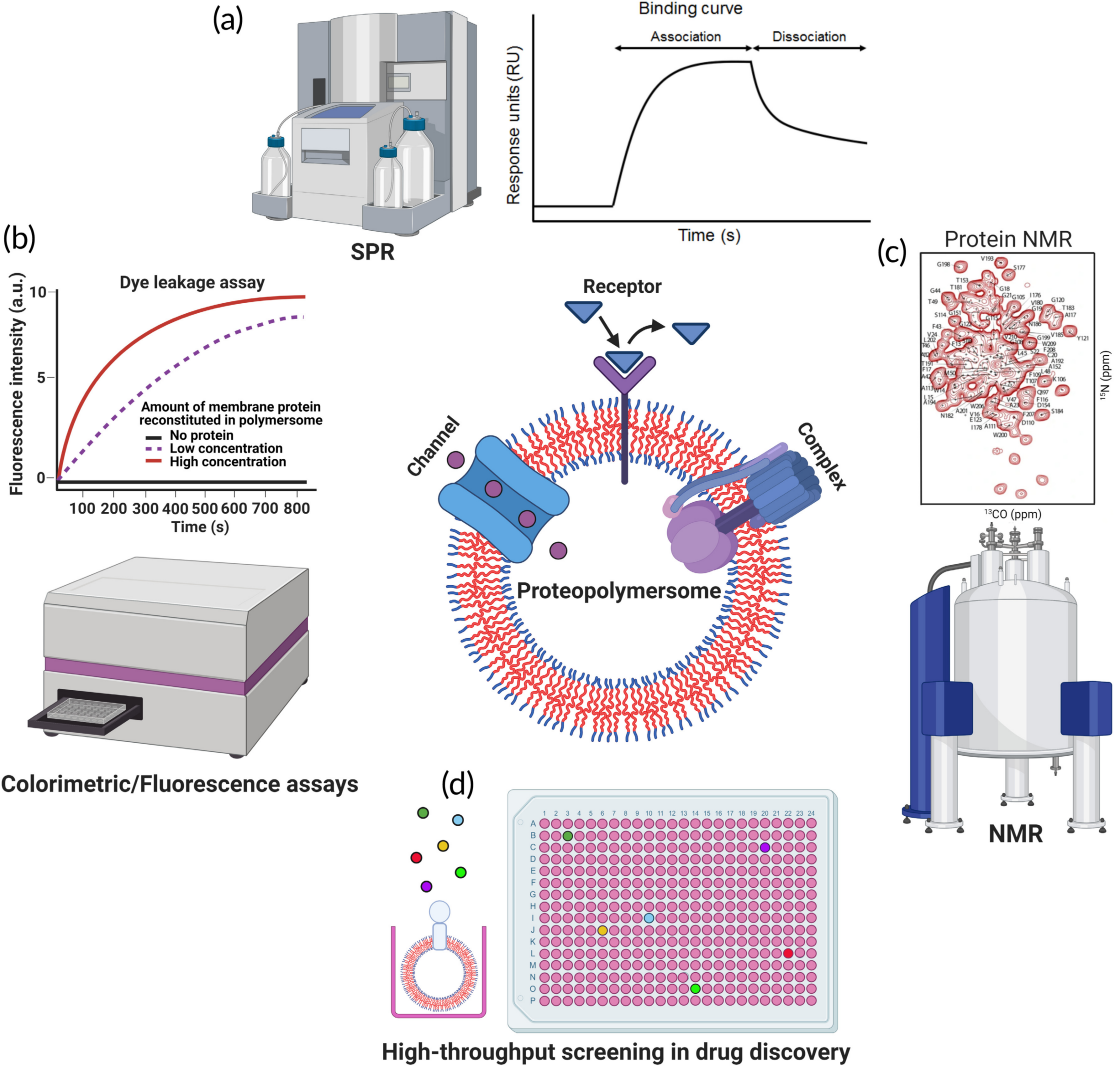
Polymersomes as platforms for MP study and drug discovery. Polymersomes, which are made up of block copolymers, can mimic biological membranes for reconstitution or incorporation of MPs, including channels, receptors, and protein complexes to form proteopolymersomes (center). Proteopolymersomes can be used to study the structure–function relationship of MPs including the characterization of (a) receptor‐ligand binding through the use of surface plasmon resonance (SPR),^33^ (b) channel transport function through conducting fluorescent dye leakage assay, and (c) MP structure by nuclear magnetic resonance (NMR).*Source*: Figure 1c is reproduced with permission from reference 34, Copyright 2018, Springer Nature. (d) Proteopolymersomes can also be used in high‐throughput screening (HTS) for drug discovery to identify modulators of MPs. Schematics were created with BioRender.com.

Apart from finding a suitable membrane support, it is crucial to ensure that the inserted MPs are folded in the correct orientation and maintain their biological functions, in order to facilitate further characterizations of these MPs.^14^, ^15^ Hence, it is imperative to optimize chemical constituents used in the formation of polymersomes or hybrid polymer‐lipid systems,^25^, ^26^, ^27^, ^28^, ^29^, ^30^ MP production methods, and parameters used in the reconstitution process.^17^, ^31^, ^32^ The reconstitution process plays a key role in determining the efficiency of reconstitution, the quality of the inserted MPs, as well as the resolution and capacity of the methods used to study these MPs.^17^, ^31^ In this article, we will review the use of polymersomes in MP structural and functional studies, as well as their translational application in high‐throughput screening (HTS) for drug discovery (Figure 1). We start by introducing the synthesis and characterization of polymersomes and methods of MP reconstitution to form proteopolymersomes. We then summarize the use of proteopolymersomes in studying both the structures and functions of channel proteins, MP complexes, and membrane receptors. Additionally, we provide a comprehensive list of factors affecting the efficiency of MP insertion and the quality of the inserted MPs. Finally, we discuss the feasibility and current applications of proteo‐nano‐vesicles in HTS. We conclude by providing future prospects in using polymersomes to engineer artificial cells as well as laying out a roadmap with recommendations for using proteopolymersomes in drug discovery pipeline.

## 2 SYNTHESIS AND CHARACTERIZATION OF POLYMERSOMES

**FIGURE 2:**
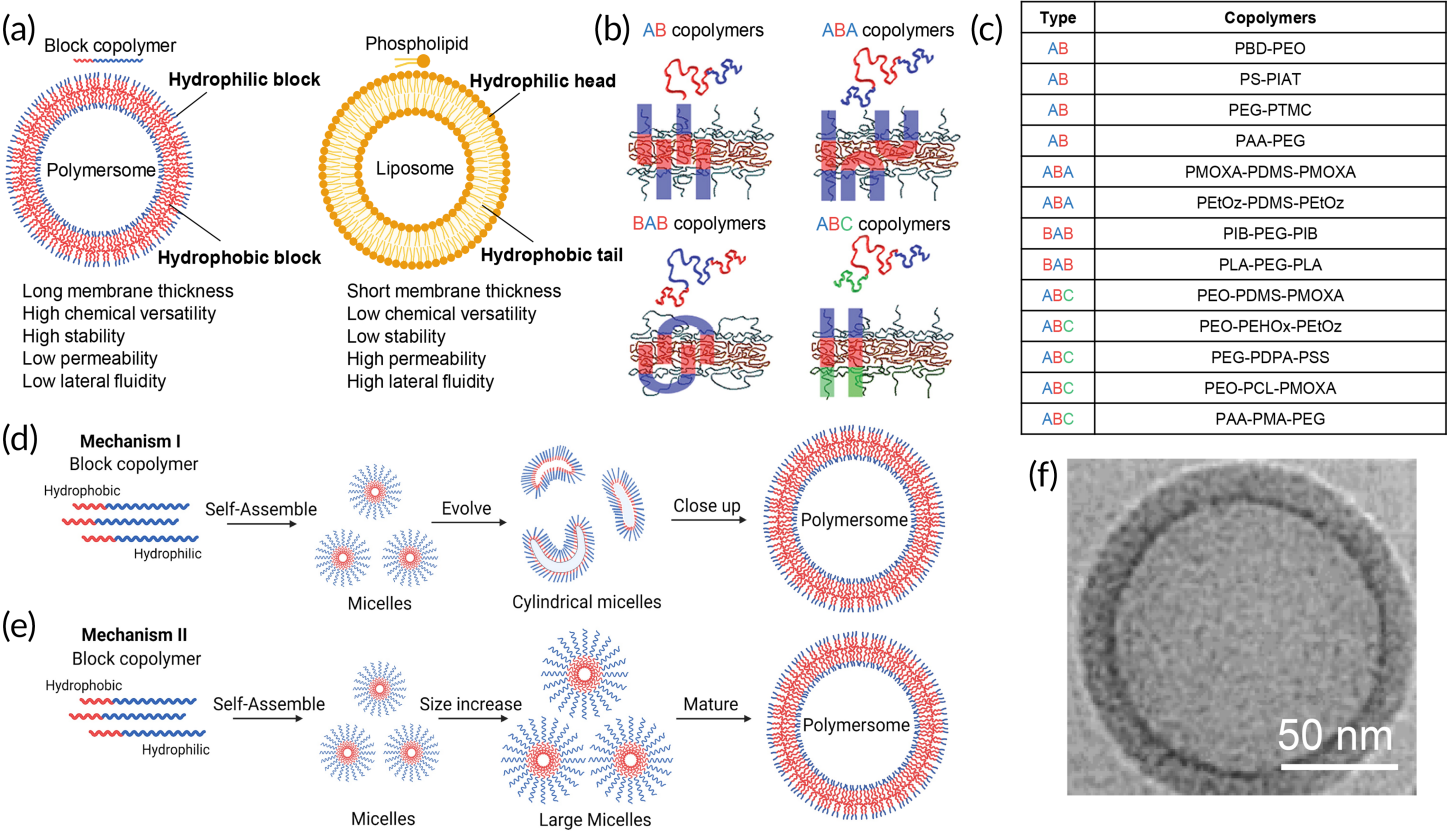
Properties of polymersomes and their formation mechanisms and characterization. (a) Comparison of vesicle properties between polymersomes and liposomes.^20^ (b) Polymersomes are formed by self‐assembly of block copolymers into a vesicular structure. Various compositions of diblock copolymers (AB) and triblock copolymers (ABA, BAB, and ABC) are used in the formation of polymersomes. *Source*: Reproduced with permission from reference 46, Copyright 2012, Elsevier. (c) A list of chemical constituents of diblock and triblock copolymers used in polymersome synthesis. (d,e) Schematics of two different proposed mechanisms for polymersome formation where (d) spherical micelles are first formed from the self‐assembly of block copolymers, which are then further self‐assembled into micelles with cylindrical or disk morphologies that can wrap around to form a vesicular shape; and (e) small spherical micelles are formed from rapid self‐assembly of block copolymers which then grow into larger micelles. *Source*: Figure 2d,e is modified and reproduced with permission from reference 44, Copyright 2011, Springer Nature. (f) Cryo‐TEM images of polymeromes formed by PEO‐PBD copolymer. The hydrophobic cores of PBD are the darker areas. Scale bar represents 50 nm. *Source*: Modified and reproduced with permission from reference 47, Copyright 2002, ACS Publications. Schematics were created with BioRender.com.

Polymersomes are spherical nanovesicular systems with polymer shells of 5–50 nm in thickness and are formed by the self‐assembly of amphiphilic block copolymers.^35^, ^36^, ^37^, ^38^ The polymersome membrane provides a physical barrier that isolates the encapsulated materials from external biological environment, while allowing controlled release or exchange of biological molecules due to the presence of a concentration gradient. A major difference between polymersomes and liposomes lies in the chemical versatility to control the thickness of the membranes where liposomes are limited to a membrane thickness of up to 5 nm, while polymersomes can have membrane thickness of up to 50 nm, depending on the type of block copolymers used.^24^ This suggests that polymersomes could potentially accommodate larger and higher amounts of MPs than liposomes, although it is important to consider the hydrophobic mismatch that might be present during MP insertion.^24^ Due to the higher molecular weight of constituent block polymers and the potential of forming cross‐linking structures through UV irradiation,^39^, ^40^ polymersomes usually have enhanced mechanical properties,^41^, ^42^ higher stability,^43^, ^44^ lower dissociation rates, lower permeability,^44^ and limited leakage^45^ compared to liposomes (Figure 2a).^20^, ^22^ Furthermore, their dense hydrophilic polymer brush‐like coronas increases their resistance to degradation and have longer circulation half‐lives in vivo.^48^

### 2.1 Types of copolymers used in polymersome synthesis

Diblock (AB) and triblock (ABA, BAB, and ABC) copolymers^35^, ^36^, ^37^, ^38^ are usually used in polymersome synthesis, with A and C being the hydrophilic blocks and B being the hydrophobic block (Figure 2b,c).^46^, ^47^ Control over the polymer block length and the hydrophilic to hydrophobic block ratio allow for tuning of membrane thickness, morphology, rigidity, and permeability of the polymersome.^23^, ^37^, ^49^, ^50^

#### 2.1.1 Diblock copolymers

The most commonly used diblock polymers is poly(butadiene)‐*b*‐poly(ethylene oxide) (PBD‐PEO)‐based.^47^, ^49^, ^51^ Their ability to provide more fluidity over other diblock copolymers make them suitable for studying membrane receptors.^52^, ^53^ Polystyrene‐*b*‐poly(isocyanoalanine[2‐thiophen‐3‐yl‐ethyl]amide) (PS‐PIAT) diblock copolymers self‐assemble into an intrinsically porous bilayer,^54^ and have been used to overcome the issue of lower permeability in polymersomes, allowing the function of larger channel or pore‐forming proteins to be tested. Other forms of diblock polymers that have been used in MP studies include poly(ethylene glycol)‐*b*‐poly(trimethylene carbonate) (PEG‐PTMC)^55^ and poly (methyl acrylate)‐*b*‐poly(ethylene glycol) (PAA‐PEG).^56^

#### 2.1.2 Triblock copolymers

Poly(2‐methyloxazoline)‐poly(dimethylsiloxane)‐poly(2‐methyloxazoline) (PMOXA‐PDMS‐PMOXA)^57^, ^58^ is the most commonly used triblock (ABA) polymer in polymersome synthesis for MP studies. ABA polymers can change their conformation to adapt to the MP length to overcome hydrophobic mismatch, as demonstrated in reconstitution of outer membrane porin F (OmpF) protein in PMOXA‐PDMS‐PMOXA^59^ and ATP synthase, or bacteriorhodopsin (BR) reconstitution in poly(2‐ethyl‐2‐oxazoline)‐*b*‐poly(dimethylsiloxane)‐*b*‐poly(2‐ethyl‐2‐oxazoline) (PEtOz‐PDMS‐PEtOz).^60^, ^61^, ^62^ To create a polymeric nanocompartment with low permeability, polyisobutylene‐polyethylene glycol‐polyisobutylene (PIB‐PEG‐PIB) (BAB) with the PIB unit being impermeable to many molecules,^63^ has been used in the formation of polymersomes with the insertion of an *Escherichia coli* (*E. coli*) outer MP.^64^ Poly(lactic acid)‐poly(ethylene glycol)‐poly(lactic acid) (PLA–PEG–PLA) is another type of BAB polymer, which has been used to synthesize polymersomes as nanocarriers for delivery of hydrophilic and hydrophobic drugs.^65^

To account for the membrane asymmetry in lipid composition, poly(ethylene oxide)‐*b*‐poly(dimethylsiloxane)‐*b*‐poly(2‐methyloxazoline) (PEO‐PDMS‐PMOXA) (ABC) is used.^66^, ^67^ ABC polymers can adopt a mixture of hairpin or transmembrane orientations due to steric hindrance and are useful for MP study as they can change their chemical composition to influence the orientation of the inserted integral proteins upon the application of external fields such as electric fields to its membrane leaflets.^68^ Recently, an one‐pot synthesis method of a new ABC triblock terpolymer, poly(ethylene oxide)‐*block*‐poly(2‐(3‐ethylheptyl)‐2‐oxazoline)‐*block*‐poly(2‐ethyl‐2‐oxazoline) (PEO‐PEHOx‐PEtOz), using sequential microwave‐assisted polymerization has been reported.^69^ The asymmetry of the formed polymersomes can be adjusted by varying the ratio of PEO to PEtOz and potentially be used for directed insertion of MPs. In another study, poly(ethylene glycol)‐poly(diisopropylaminoethyl methacrylate)‐*b*‐poly(styrenesulfonate) (PEG‐PDPA‐PSS) has been used for directed insertion of proteorhodopsin (PR).^70^ Other types of ABC polymers, including poly(ethylene oxide)‐*b*‐polycaprolactone‐*b*‐poly(2‐methyl‐2‐oxazoline) (PEO‐PCL‐PMOXA)^71^ and PAA‐PMA‐PEG^56^ have also demonstrated success in forming polymersomes and may offer new avenues for MPs study in novel applications.

### 2.2 Synthesis of polymersomes

There are two different proposed mechanisms for the formation of polymersomes where (i) spherical micelles are first formed from the self‐assembly of block copolymers, which are then further self‐assembled into micelles with cylindrical or disk morphologies that can wrap around to form a vesicular shape (Figure 2d); and (ii) small spherical micelles are formed from rapid self‐assembly of block copolymers, which then grow into larger micelles and polymersomes (Figure 2e).^72^ Specifically, polymersomes can be synthesized from different copolymers via solvent‐displacement, polymer film rehydration, solid rehydration, or electroformation techniques.^43^, ^67^, ^73^ In solvent displacement method, the polymer is dissolved in a suitable organic solvent and added dropwise to an aqueous buffer and stirred vigorously to form an emulsion. While being a simple and fast method, the polydispersity of polymersome sizes is high,^74^ and residual organic solvents may denature most amphiphilic MPs and result in low reconstitution efficiency.^75^ To overcome the use of organic solvents, polymer rehydration technique has been developed, where the polymer solution is first dried to remove traces of organic solvents before rehydration in aqueous buffers. Polyethyleneoxide‐polyethylethylene (PEO‐PEE)‐based polymersomes generated using the polymer film rehydration method yields small polymersomes with a size of about 100 nm but with a broad size distribution.^45^ In solid rehydration, the polymer is made into bulk powder form before rehydration in aqueous buffers. However, it requires stronger and longer agitation time for complete rehydration.^45^ Electroformation is another method commonly used to synthesize PMOXA‐PDMS‐PMOXA and PB‐PEO polymersomes,^76^, ^77^ but this method results in polymersomes in a larger size range of 10–40 μm.^78^ Other techniques include 3D soft‐confined solvent annealing,^79^ droplet microfluidic that have been used to produce PEG–PLA‐based polymersomes,^80^ and gel‐assisted rehydration where polymer solutions are spread across dehydrated agarose films and subsequently rehydrated in aqueous buffers.^81^

### 2.3 Characterization of polymersomes

The hydrodynamic radius, size distribution, and morphology of the formed polymersomes can be characterized by dynamic light scattering (DLS), static light scattering (SLS), optical microscopy, and transmission electron microscopy (TEM).^82^ High‐throughput scattering methods such as combinatorial small‐angle X‐ray scattering (SAXS) or wide‐angle x‐ray scattering (WAXS) can provide information about structural features of colloidal size, including membrane bilayer thickness and internal structure.^83^ The small‐angle neutron scattering (SANS) technique can study the morphology and thermodynamics of polymer blends and copolymers in polymersomes, as well as the polymersome structure.^84^ Optical microscopy can only resolve polymersomes larger than 1 μm in diameter,^85^ while higher resolution imaging tools such as TEM, cryo‐TEM, and freeze fracture cryo‐scanning electron microscopy (FF‐Cryo‐SEM) are able to give about a 1000‐fold increase in resolution and a 100‐fold increase in depth of field.^85^ In particular, cryo‐TEM can avoid the drying process associated artifacts in electron microscopy sample preparation and can provide the information regarding the size, morphology, and bilayer thickness of polymersomes (Figure 2f).^83^ Atomic force microscopy (AFM) can also be used to characterize the mechanical properties of polymersomes.^83^

## 3 STRATEGIES FOR MP INSERTION TO FORM PROTEOPOLYMERSOMES

The reconstitution or insertion of MPs in polymersomes has emerged as a powerful tool in studying the structure and functionality of MPs.^86^ To retain the structural integrity of MPs and confer their biological functionalities, MPs have to be preserved in amphiphilic environment similar to their native environment such as the use of detergents to prevent denaturation. The protein–detergent–membrane interaction play a key role in MP insertion, which is affected by the different methods of protein production and purification, the type and amount of detergents used, and the different physicochemical properties of polymersomes, including their fluidity and flexibility. MPs can be reconstituted via three major methods: (1) cell‐based protein production and detergent mediated reconstitution,^87^ (2) cell‐free co‐translational protein production and direct incorporation,^53^ and (3) reconstitution by destabilization of vesicles or supported planar bilayer membranes.^88^, ^89^, ^90^, ^91^, ^92^, ^93^ Following reconstitution, purification steps such as dialysis, gel filtration or size exclusion chromatography (SEC), centrifugation, and bio‐beads aided procedures should be carried out to remove excess detergents and other reagents to enhance the formation of stable proteopolymersomes.

### 3.1 Cell‐based protein production and detergent mediated reconstitution

**FIGURE 3:**
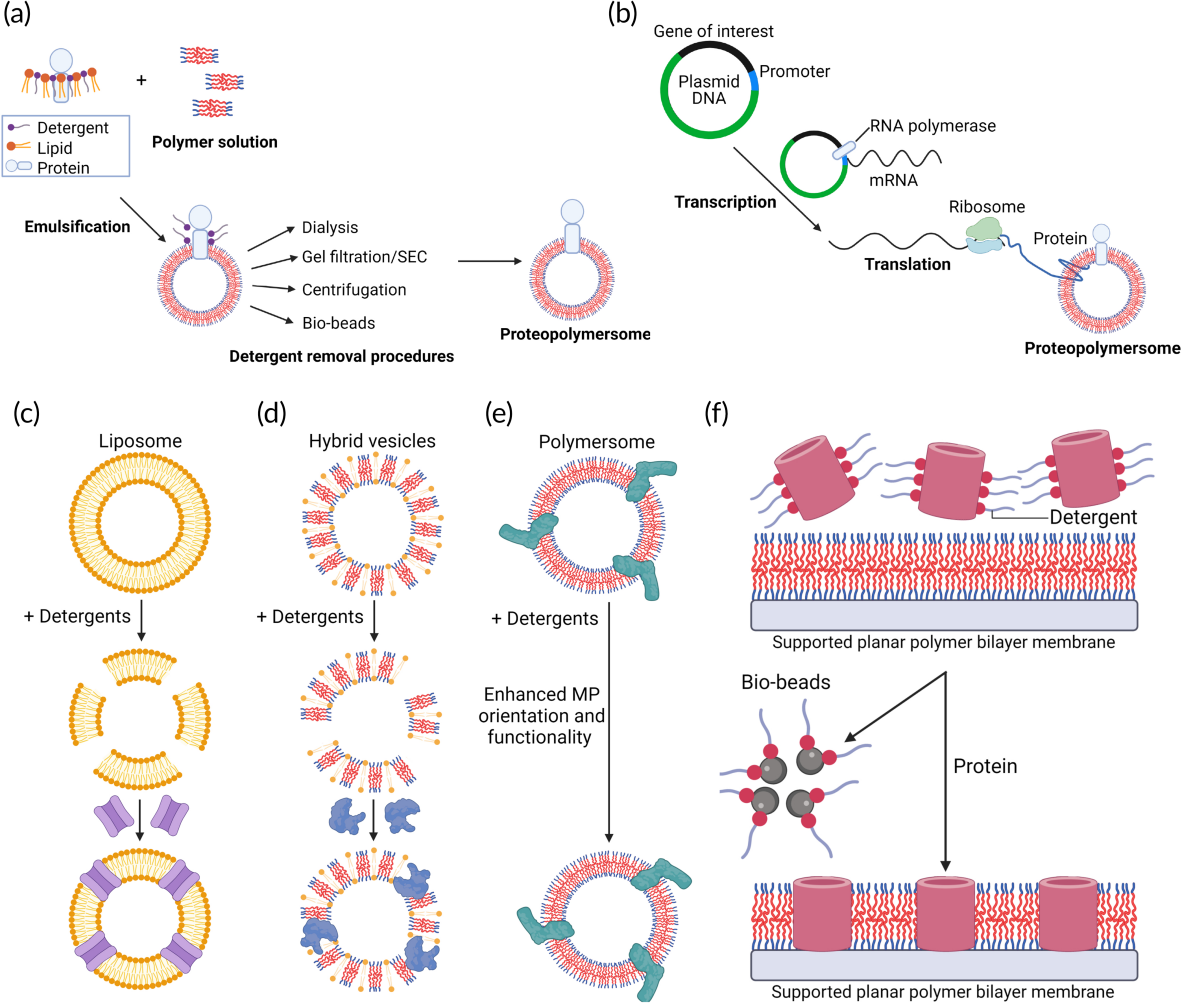
MP insertion strategies to form proteopolymersomes. (a) Detergent‐mediated reconstitution of MPs into polymersomes. MPs from native membranes are purified, solubilized, and stabilized by detergents. The MP solution is then mixed with polymers dissolved in organic solvent to form an emulsification with a mixture of polymer–protein–detergent micelles. When detergent is removed from the micellar solutions via procedures such as dialysis, gel filtration/SEC, centrifugation or with the use of bio‐beads, MPs are reconstituted into vesicles forming proteopolymersomes. *Source*: Modified and reproduced with permission from reference 87, Copyright 2002, SciELO. (b) Spontaneous incorporation of MPs into polymersome to form proteopolymersomes through cell‐free protein synthesis by adding complementary DNA encoding the protein of interest and polymersomes directly to an in vitro expression mixture, including RNA polymerase and ribosome. *Source*: Modified and reproduced with permission from reference 53, Copyright 2012, John Wiley and Sons. (c–e) Vesicle destabilization by detergents in (c) liposome with reconstitution of NorA multidrug efflux transporter as an example,^88^ (d) hybrid vesicle made of lipids and polymers with reconstitution of Cyt‐bo3 ubiquinol oxidase as an example,^89^ and (e) polymersome with enhanced orientation and improved functionality of NADH:ubiquinone oxidoreductase (Complex I) as an example.^90^ (f) Destabilization of supported planar polymer bilayer membrane by bio‐beads for MP reconstitution with functional insertion of M1oK1 as an example. *Source*: Modified and reproduced with permission from reference 91, Copyright 2014, Elsevier. Schematics were created with BioRender.com.

The recombinant MPs are first purified from plasmid transformed bacteria cultures, and the purified MPs are solubilized with detergents and emulsified with excess polymers via self‐assembly, followed by detergent removal (Figure 3a).^32^, ^94^ The addition of detergents allows for ease of MPs solubilization and keeps them in a native environment to facilitate MPs folding and stabilization. Upon protein reconstitution, the detergent molecules need to be removed to aid in the formation of stable vesicles, and residual detergent may also inhibit protein activity.^94^ Multiple MPs have been reconstituted into polymersomes through this approach with common detergent removal methods including dialysis,^95^ gel filtration or SEC,^86^, ^87^ centrifugation,^52^ or bio‐beads aided procedures (Table 1).^86^, ^87^ In the dialysis method, the MPs and polymersome emulsion are dialyzed against a larger volume of buffer to remove the excess detergents.^95^ For gel filtration or SEC‐mediated detergent removal, the MP‐polymersome solution is passed through a gel‐exclusion column which separate and elute the proteopolymersomes before the detergent. Different sized columns can be used ranging from Sephadex G25 to G200.^94^ This technique has the advantage of being rapid but can lead to a broader size distribution in proteopolymersomes. Using the centrifugation approach, the excess detergents as well as free MPs are filtered through centrifugal filtration cartridges of a certain molecular weight cut‐off.^90^ For bio‐beads mediated detergent removal, the beads are used to physically adsorb and sequester excess detergents, where the detergent‐bound beads can subsequently be removed by centrifugation or filtration.^94^ The choice of detergent removal method and its efficiency are dependent on the type of detergent used during the MP reconstitution process.^94^, ^114^ Detergents with a high critical micelle concentration (CMC), such as cholate and octyl glucoside, tend to form smaller micelles and make them easier to remove via the process of dialysis or by SEC.^94^, ^114^ Detergents with a lower CMC, such as Triton‐X 100 which forms larger micelles, are less likely to be removed by dialysis or SEC and hence are more often removed via bio‐beads aided process.^94^, ^114^ Some limitations associated with cell‐based protein production or MP overexpression are low yield, cell cytotoxicity, protein aggregation, and misfolding, which can in turn result in polymer membrane overcrowding.^115^

**TABLE 1:** List of proteopolymersomes based membrane protein studies

Membrane transport proteins	Block copolymers	Protein production	Insertion method; purification method	(A) Proteopolymersome characterization	References
				(B) MP structural studies	
				(C) MP functional studies	
Outer membrane protein F (OmpF)	PMOXA–PDMS–PMOXA	Cell based	Detergent mediated; gel filtration/SEC	(A) Cryo‐TEM, DLS, SLS, TEM, AFM	26, 96, 97
				(B) N/A	
				(C) Iodometry to monitor ampicillin hydrolysis by β‐lactamase; LSM	
OmpF 6His	PMOXA–PDMS–PMOXA	Cell based	Detergent mediated; gel filtration/SEC	(A) DLS	98
				(B) CD	
				(C) Leakage assay of fluorescent dye	
OmpF‐S‐S‐CF	PMOXA_6_–PDMS_44_–PMOXA_6_	Cell based	Detergent mediated; dialysis	(A) SLS, DLS, Cryo‐TEM	99
				(B) N/A	
				(C) Fluorescence generated when using AmR as a substrate for HRP, FCS, EPR	
Aquaporins (AQPs)	PMOXA_11_–PDMS_34_	Cell based	Detergent mediated; dialysis	(A) DLS	95
				(B) N/A	
				(C) SFLS, DLS	
AQPZ	PMOXA_15_–PDMS_110_–PMOXA_15_	Cell based	Detergent mediated; bio‐beads	(A) DLS, FETEM	100
				(B) N/A	
				(C) SFLS	
AQP0	PEO_14_–PB_22_, PEO_14_–PB_22_, PMOXA_20_–PDMS_42_–PMOXA_20_ PMOXA_12_–PDMS_55_–PMOXA_12_	Cell based	Detergent mediated; gel filtration/SEC or Dialysis	(A) EM, RS	101, 102
				(B) N/A	
				(C) SFLS	
Ferric hydroxamate uptake protein component A (FhuA) FhuA Δ1–129 FhuA Δ1–160	PMOXA–PDMS–PMOXA	Cell based	Detergent mediated; bio‐beads or dialysis	(A) ITC, DLS	27, 103
				(B) CD	
				(C) FCS	
FhuA Δ1–159	PIB_1000_–PEG_6000_–PIB_1000_	Cell based	Detergent mediated; gel filtration/SEC	(A) DLS, Cryo‐TEM	64
				(B) CD	
				(C) Absorbance detection at 370 nm of TMB oxidation product when using TMB as a substrate for encapsulated HRP	
Gramicidin A (gA)	PMOXA–PDMS–PMOXA	Cell based	Detergent mediated; gel filtration/SEC	(A) TEM, SLS	104
				(B) N/A	
				(C) SFLS; Fluorescence spectroscopy on changes of the ANG‐2 dye specific for Na^+^ transport and APG‐2 dye specific for K^+^ transport	
Ionomycin	PMOXA_6_–PDMS_44_–PMOXA_6_	Cell based	Detergent mediated; gel filtration/SEC	(A) TEM	105
				(B) N/A	
				(C) SFLS; Fluorescence spectroscopy on changes in the calcium sensitive ACG dye due to influx of Ca^2+^ ions	
KcsA	PMOXA–PDMS–PMOXA	Cell based	Detergent mediated; dialysis	(A) FCS	59
				(B) Z‐scan fluorescence correlation spectroscopy	
				(C) N/A	
Maltoporin (LamB)	PMOXA–PDMS–PMOXA	Cell based	Detergent mediated; gel filtration/SEC	(A) Langmuir trough	28
				(B) N/A	
				(C) Fluorescence spectroscopy monitoring the change in fluorescently labeled DNA released into the vesicle	
Nucleoside‐specific porin (TsX)	PMOXA_20_–PDMS_54_ PMOXA_20_	Cell based	Detergent mediated; gel filtration/SEC	(A) DLS, Gel electrophoresis	106
				(B) N/A	
				(C) Fluorescence due to hydrolysis of prodrug 2‐fluoroadenosine to 2‐fluoroadenine	
Large conductance mechano‐sensitive ion channel (MscL)	Hybrid vesicles: (a) 1,2‐dioleoyl‐sn‐glycero‐3‐phosphocholine (DOPC) (b) PEO_9_‐b‐PBD_12_; PEO_14_‐b‐PBD_22_; PEO_24_‐b‐PBD_36_	Cell free	Co‐translational incorporation; gel filtration/SEC	(A) Western blotting	51
				(B) mEGFP fluorescence due to proper folding	
				(C) Leakage assay of fluorescent dye	
α‐Hemolysin	PBD‐PEO	Cell free	Co‐translational incorporation; centrifugation	(A) SEM	52
				(B) N/A	
				(C) Leakage assay of fluorescent dye	
NADH: ubiquinone oxidoreductase (Complex I)	PMOXA_(9–64)_–PDMS_(23–165)_–PMOXA_(9–64)_	Cell based	Detergent mediated with vesicle destabilization; bio‐beads	(A) EPR, BCA	90
				(B) N/A	
				(C) NADH/Ferricyanide or NADH/Decylubiquinone or NADH:Ubiquinone 2/AQ oxido‐reductase activity assay; Complex I inhibition assay	
F_o_F_1_‐ATPase and BR	PEtOz−PDMS−PEtOz	Cell based	Detergent mediated; dialysis	(A) TEM	61, 62, 107
				(B) N/A	
				(C) Production of photoinduced electrochemical proton gradient; ATP synthesis activity	
Proteorhodopsin (PR)	PEG–PDPA–PSS	Cell based	Detergent mediated; centrifugation	(A) TEM	70
				(B) PR would orientate with negatively charged PSS	
				(C) Light‐activated pH changes	
Proteorhodopsin (PR)	PS‐*b*‐P4MVP_2_	Cell based	Detergent mediated; centrifugation	(A) TEM	108
				(B) SAXS, RS, ssNMR	
				(C) Time‐resolved visible spectroscopy (flash photolysis)	
Cytochrome bo3 (Cyt‐bo3)	Hybrid vesicles: (a) PBd_22_‐*b*‐PEO_14,_ (b) POPC	Cell based	Detergent mediated or vesicle destabilization; bio‐beads	(A) DLS and TEM	89
				(B) N/A	
				(C) Decylubiquinone oxidation reaction initial rate	
NaAtm1 P‐glycoprotein (PgP)	Hybrid vesicles: (a) Palmitoyl‐oleoyl‐phosphatidylcholine, (b) PBD‐PEO	Cell based	Detergent mediated; bio‐beads	(A) Flotation in a sucrose density gradient	30
				(B) N/A	
				(C) Passive permeability to a fluorescent probe	

Membrane receptors	Block copolymers	Protein production	Insertion method; purification method	(A) Proteopolymersome characterization	References
				(B) MP structural studies	
				(C) MP functional studies	
Dopamine receptor D2 (DRD2)	PBD–PEO PMOXA–PDMS–PMOXA	Cell free	Co‐translational incorporation; centrifugation	(A) Western blotting	53
				(B) Conformational antibody binding (SPR)	
				(C) Native ligand binding and replacement assay (compete dye‐labeled ligand with unlabeled ligand)	
C‐X‐C chemokine receptor type 4 (CXCR4)	PB–PEO	Cell free	Co‐translational incorporation; centrifugation	(A) Western blotting	109
				(B) Conformational antibody binding (SPR)	
				(C) Native ligand binding	
Glucagon‐like peptide‐1 receptor (GLP‐1R)	PBD–PEO PMOXA–PDMS–PMOXA	Cell free	Co‐translational incorporation; dialysis	(A) Western blotting, TEM, DLS, SEC	110
				(B) Conformational antibody binding (SPR)	
				(C) Native ligand binding, radioligand saturation binding assay	
Claudin‐2 (Cldn‐2)	PBD–PEO	Cell free	Co‐translational incorporation; centrifugation	(A) SEM, Western blotting	52
				(B) Conformation antibody binding (SPR)	
				(C) N/A	
Major histocompatibility complex I (MHC‐I)	PMOXA–PDMS–PMOXA	Cell free	Co‐translational incorporation; bio‐beads	(A) Fluorescent microscopy images of antibody binding	111
				(B) Conformational antibody binding (SPR)	
				(C) T‐cell activation	
Peptide anchors	PMOXA–PDMS–PMOXA	Cell based	Detergent mediated; gel filtration/SEC	(A) SEC, confocal microscopy, tryptophan fluorescence measurements	112, 113
				(B) Intact with membrane (nonpore forming), CD	
				(C) N/A	

Abbreviations: ACG, Asante Calcium Green; AFM, atomic force microscopy; AmR, Amplex UltraRed; ANG‐2,Asante NaTRIUM Green‐2; APG‐2, Asante Potassium Green‐2; BCA, bicinchoninic acid protein assay; CD, circular dichroism; DLS, dynamic light scattering; EM, electron microscopy; EPR, electron paramagnetic resonance; FCS, fluorescence correlation spectroscopy; FETEM, field emission transmission electron microscopy; HRP, horse radish peroxidase; ITC, isothermal calorimetry; LSM, laser scanning microscopy; RS, Raman spectroscopy; SAXS, small angle x‐ray scattering; SEC, size exclusion chromatography; SFLS, stopped flow light scattering kinetics; SLS, static light scattering; SPR, surface plasmon resonance; ssNMR, solid‐state NMR spectroscopy; TEM, transmission electron microscopy; TMB, 3,3′,5,5′‐tetramethylbenzidine.

### 3.2 Cell‐free co‐translational protein production and direct incorporation

The MP of interest is expressed from a plasmid and directly incorporated into the polymersome (Figure 3b).^116^ In this method, the cDNA coding for the MP of interest and reaction mixtures containing necessary components for protein translation are added to polymersomes in solution and incubated at elevated temperatures for a few hours. A typical reaction mixture is composed of a cell extract from *E. coli*, wheat germ, or rabbit reticulocytes, containing components such as ribosomes, translation factors, aminoacyl‐tRNA synthetases, and tRNAs, which are required for production of protein.^117^, ^118^, ^119^ A more recent development is cell‐free protein synthesis using recombinant elements (PURE) system, which comprises individually purified components of the *E. coli* translation apparatus.^120^ The PURE system does not contain cell extract and results in less degradation of cDNA template as well as protein products, thereby allowing for more efficient incorporation of MPs.^120^ The cell‐free method also allows direct access to reaction conditions, where additional agents which aid the reconstitution process such as detergents or protein folding catalysts can be included.^115^ The cell‐free method overcomes the issues associated with conventional overexpression and reconstitution of MPs into membrane models, such as low protein yields, cytotoxicity, misfolding, and aggregation.^121^, ^122^ Upon reconstitution, the proteopolymersome size and morphology can be further fine‐tuned through freeze–thaw, extrusion, and sonication methods.^94^, ^114^ Polymersomes without MPs, as well as excess cell‐free expression reaction reagents, can be removed from proteopolymersomes by methods similar to detergent removal including dialysis,^110^ gel filtration or SEC,^86^, ^87^ centrifugation,^53^ and bio‐beads mediated process.^111^ A limitation of the direct incorporate approach is that the necessary posttranslational modifications, which are required for the formation of fully functional proteins may not occur, unless known enzymes responsible for these processes are added to the reaction mixture.^123^

### 3.3 Reconstitution by destabilization of vesicles or supported planar bilayer membranes

Membrane destabilization by detergents has been used to reconstitute MPs in liposomes (Figure 3c)^88^ and hybrid vesicles (Figure 3d),^89^ as wells as a way to enhance the orientation and functionality of reconstituted MPs in polymersomes (Figure 3e).^90^ While the use of detergents and their removal are also necessary in this approach, the key difference lies in the vesicles or proteo‐vesicles being formed first, followed by the addition of detergents to perturb the integrity of the vesicles to allow for solubilized MPs insertion^88^ or reorientation of the inserted MPs.^90^ Multidrug resistance (MDR) transporter NorA was incorporated in liposomes made from *E. coli* polar lipid crude extract by destabilization using detergents.^88^ Liposome destabilization was achieved by the stepwise addition of Triton X‐100 and mixed with NorA protein solution, and bio‐beads were added for detergent removal.^88^ Cytochrome bo3 (Cyt‐bo3) has been incorporated in hybrid vesicles made of PBD‐PEO and POPC using detergent‐mediated reconstitution. Hybrid vesicles are first formed by extrusion and destabilized by gradual addition of small concentrations of Triton X‐100 detergent. At the brink where the detergent started to break up the integrity of the hybrid vesicles, Cyt‐bo3 solutions were added and incorporated into the vesicles, where the excess detergents are then removed by bio‐beads.^89^ NADH:ubiquinone oxidoreductase (Complex I) was incorporated in PMOXA‐PDMS‐PMOXA polymers using detergent‐mediated reconstitution. Partial destabilization of the polymer membrane by adding Triton X‐100 detergent allows for rearrangement of the inserted Complex I to enhance its structural orientation with a considerable fraction of vesicles remained intact.^90^

Other types of membrane destabilization methods, such as voltage and bio‐beads mediated destabilization, have been used to reconstitute MPs on supported planar lipid or polymer bilayer membranes. Bio‐beads mediated MP insertion has been used for the insertion of MloK1, a cyclic nucleotide‐modulated potassium channel from Mesorhizobium loti, into supported PDMS‐PMOXA‐based polymeric membranes (Figure 3f).^91^ To achieve functional insertion of M1oK1, both the protein and the polymer membrane were destabilized by bio‐beads. The bio‐beads provided the driving force for the insertion of the MP into the polymer membrane. The conductance across M1oK1 increased only when protein reconstitution was carried out in the presence of bio‐beads.^91^ Voltage destabilization is another approach that has been suggested with the insertion of α‐hemolysin into supported planar polymer membranes made of PB‐PEO diblock copolymers as an example.^92^, ^93^

Cell‐based protein production followed by detergent‐mediated reconstitution has been the predominantly used method in MP insertion. The adoption of the cell‐free co‐translational incorporation approach, which overcomes limitations in cell‐based protein production, has been on a rise. The membrane destabilization method is still largely limited to MP reconstitution in liposomes, hybrid vesicles or planar membrane bilayers. Regardless of the methods used, reproducibility and predictability are two important requirements to fulfill in the engineering of proteopolymersomes to allow for accurate acquisition of biological information related to the MPs of interest and their applications such as in engineering of artificial cells and drug discovery. In general, the proteopolymersomes formed should have bilayer thickness that match MP hydrophobic domain, high mechanical strength, good stability, and conformation flexibility to adapt to MP insertion and functionality.

## 4 MEMBRANE TRANSPORT PROTEINS

Membrane transport proteins are MPs that play important roles in maintaining the physiological function of cells. There are two different types of transport (passive and active) across cell membranes. Passive transport requires no energy input as transport follows a concentration gradient and examples include channel proteins.^124^ In contrast, active transport requires energy, most commonly from ATP hydrolysis by primary active transporters, which include proton pumps. Active transport is used to carry substances into a cell against the concentration gradient.^125^ Liposomes have been used to study membrane transport proteins, in particular channel proteins; however, their highly fluid and leaky nature hinders the retention of molecules, often resulting in inaccurate measurement of these protein functions.^20^, ^125^ Polymersomes can overcome these issues with their low passive permeability to low‐molecular‐weight solute,^44^ and have been used widely by researchers to reconstitute and incorporate channel proteins or porins.^99^ Apart from studying the functional activity of channel proteins, the activity of protein complexes can also be modeled and studied with proteopolymersomes. These complexes include primary active transporters and MP coupling systems such as NADH:ubiquinone oxidoreductase (Complex I), F_0_F_1_‐ATPase, and proton pumps. We have summarized the various types of channel proteins for passive transport and protein complexes for active transport studied in polymersomes (Table 1).

### 4.1 Channel proteins for passive transport

#### 4.1.1 OmpF

**FIGURE 4:**
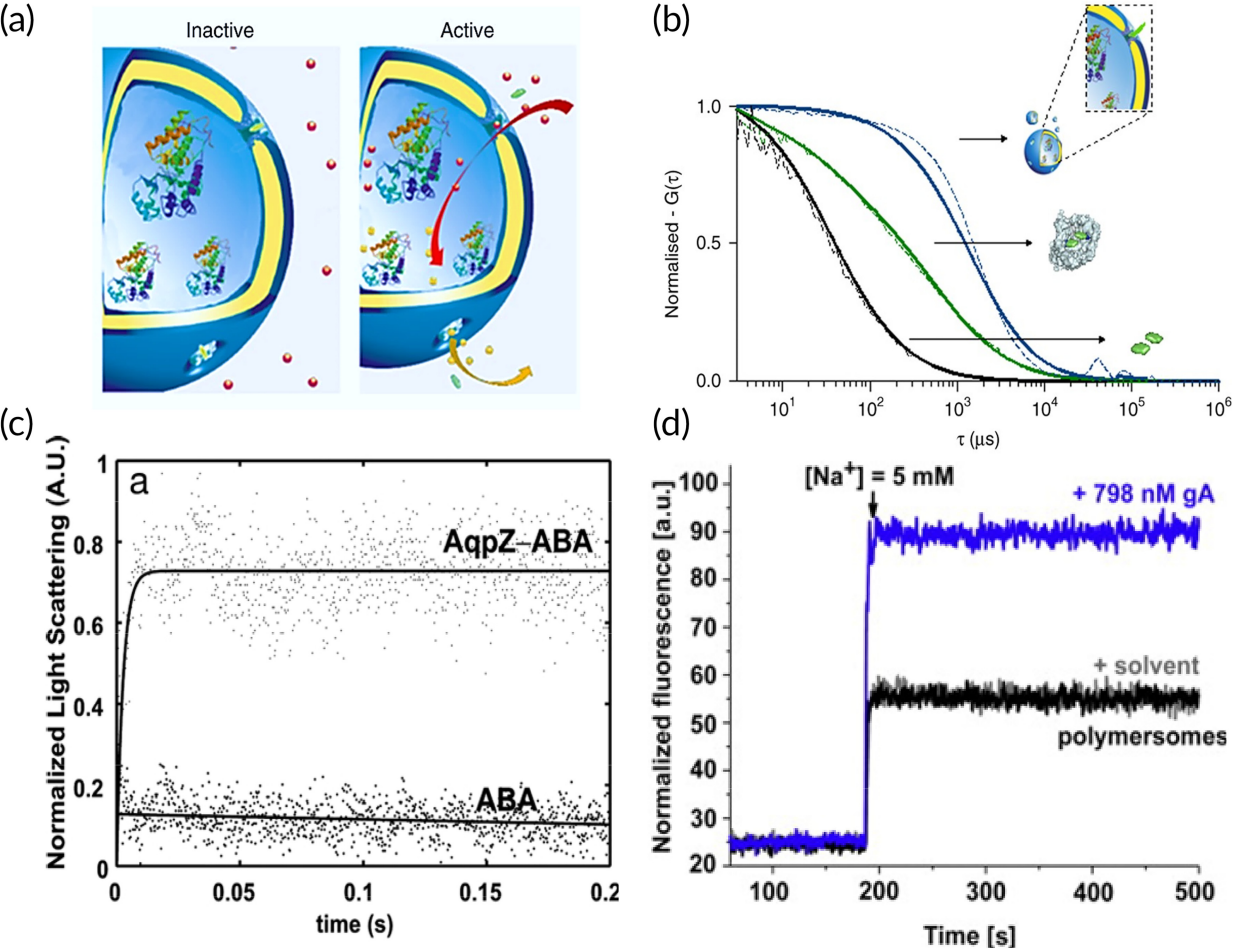
Structural and functional studies of channel proteins with passive diffusion using polymersomes. (a) Schematic of polymersomes with channel proteins reconstituted to allow passage of solutes and selective permeability of ions when the channel proteins are active. Example shown is an OmpF proteopolymersome. (b) Insertion of SAMSA fluorescein (SAMSA‐CF) conjugated OmpF with K89C and R270C double mutations through disulfide bonding (OmpF‐S‐S‐CF) was evaluated with fluorescence correlation spectroscopy. Reconstitution of OmpF‐S‐S‐CF into polymersome (blue) increases protein diffusion time, compared to OmpF‐S‐S‐CF in surfactant (1% octyl‐glucopyranoside/1% OG) (green), and SAMSA‐CF only control (black). Dotted line refers to experimental autocorrelation curves and solid line refers to fitted curve. *Source*: Figure 3a,b is reproduced with permission from reference 99, Copyright 2018, Springer Nature. (c) Stopped‐flow light‐scattering experiment to characterize vesicle permeability in aquaporin Z (AqpZ) proteopolymersomes. The initial rise rates are used to calculate the permeability and there is an increase in relative light scattering when AqpZ is reconstituted in the polymersomes. *Source*: Reproduced with permission from reference 101, Copyright 2007, National Academy of Sciences, USA. (d) Measurements of Na^+^ influx in ANG‐2 (Na^+^ specific dye) loaded polymersomes before and after reconstitution of gramicidin A (gA). The presence of gA allows for higher influx of Na^+^ ions into the polymersomes, resulting in an increase in fluorescence intensity of the ANG‐2. *Source*: Reproduced with permission from reference 104, Copyright 2015, Elsevier

The outer membrane protein F (OmpF) is a MP that functions as a passive diffusion channel in *E. coli* and assembles to form a highly stable trimer in membranes. OmpF functions as the main route of outer membrane penetration for many antibiotics, hence studying its structure and function can be of clinical importance in determining bacterial resistance mechanisms and therapeutic advancements.^126^ OmpF is the first MP successfully reconstituted with full functionality into PMOXA–PDMS–PMOXA membranes.^25^, ^26^, ^96^, ^97^, ^106^ The OmpF reconstitution efficiency is increased with homogenous distribution of MPs and polymers coupled with slow controlled removal of surfactants.^26^ The successful passage of antibiotics, such as ampicillin, demonstrates the functional reconstitution of OmpF in polymersomes.^25^, ^26^ OmpF function has also been determined through monitoring the conversion of passaged substrates with no antibacterial activity into substrates with bacterial activity or antibiotics by the encapsulated penicillin acylase enzyme.^97^ OmpF containing double mutants (K89C and R270C) with SAMSA fluorescein conjugation through disulfide bonding termed as OmpF‐S‐S‐CF is reconstituted into PMOXA‐PDMS‐PMOXA polymersomes via the rehydration method (Figure 4a).^99^ The successful insertion of OmpF into polymersomes is evaluated using fluorescence correlation spectroscopy (FCS) to determine whether there is an increase in diffusion time (Figure 4b),^99^ or by electron paramagnetic resonance (EPR) which has a broad spectrum, indicative of low mobility due to successful MP reconstitution. The protein functions are determined via encapsulating horse radish peroxidase (HRP) in polymersomes and monitoring for changes in fluorescence due to the formation of resorufin‐like product upon successful diffusion of Amplex UltraRed (AmR), a substrate for HRP. The reconstituted proteopolymersomes show good biocompatibility in a zebrafish embryo model, demonstrating potential use of these polymersomes as cellular implants in living organisms.^99^ Other modified OmpF such as OmpF 6His has also been successfully reconstituted in PMOXA–PDMS–PMOXA polymersomes.^98^ The structure of the OmpF 6His is determined with circular dichroism (CD) in solution, which indicates that OmpF 6His adopts a β‐barrel stable structure in proteopolymersome. Functional reconstitution of OmpF 6His is determined through measuring a significant release of encapsulated acridine orange outside of the proteopolymersomes when the pH was increased from 5 to 7 across the OmpF, which allows for protons to pass through and result in changes in acridine orange.^98^

#### 4.1.2 Aquaporins

Another widely studied class of channel proteins is the aquaporins (AQPs), which are water channels that can mediate bidirectional, transmembrane water flow in the presence of an osmotic gradient. Its dysfunction is associated with multiple human diseases, such as glaucoma, cancer, epilepsy, and obesity.^127^ Several AQPs have been reconstituted into PMOXA‐PDMS copolymer‐based polymersomes via detergent‐mediated reconstitution including AQP1‐5, which are highly specific for water and AQP3, 7, 9, and 10, which mediate glycerol flux.^95^ The functionality of reconstituted AQPs as solute transporters of water or glycerol is studied with stopped flow light scattering kinetics, where a hyperosmotic gradient is first imposed across the membrane of the AQP proteopolymersomes, and then a hypertonic gradient is applied. Outflow from the polymersome results in faster shrinking and increase in light scattering, indicating higher water permeability as a result of functional AQP reconstitution.^95^ Aquaporin Z (AQPZ), which can maintain water permeability while retaining uncharged solutes (i.e., glucose, glycerol, salt, and urea), is reconstituted in PMOXA‐PDMS‐PMOXA polymersomes, where it shows 90 times higher water permeability than polymersomes without AQPZ insertion, as well as high rejection rates of salt, glycerol, and urea (Figure 4c).^101^ However, AQPZ incorporation has a limiting concentration at a protein‐to‐polymer ratio of 1:50, where a further increase in protein concentration decreases water permeability instead of enhancing it.^101^ This limit in ratio could be due to the method of reconstitution used, where a higher detergent concentration used in the AQPZ stock solution can lead to reduced AQPZ reconstitution efficiency. This can be overcome by using slow detergent removal methods or other reconstitution methods.^101^

In a similar study, AQPZ is reconstituted into disulfide‐functionalized PMOXA‐PDMS‐PMOXA copolymer via film rehydration technique, and the vesicle shrinkage or permeability is determined to be 4680 μm/s.^100^ Further studies show that AQPZ water permeability can be improved when reconstituted in PMOXA‐PDMS‐PMOXA membranes with a larger hydrophobic thickness, due to a decrease in Arrhenius activation energies for water transport across the AQPZ.^128^ For structural studies of AQPZ, SAXS has been used to determine AQPZ structure that has been reconstituted in PBD‐PEO polymersomes with different molecular weights. SAXS indicates that AQPZ reconstitution in PB_45_‐PEO_14_ leads to a minor difference in average hydrophobic vesicle wall thickness, which could indicate a dimpling or puckering of polymers close to the incorporated AQPZs. On the other hand, in PB_33_‐PEO_18_, micelle‐formation tendency is reduced when AQPZ is incorporated.^129^ The lens specific water channel aquaporin 0 (AQP0) was reconstituted in PEO‐PB and PMOXA‐PDMS‐PMOXA polymersomes with varying copolymer block lengths, where the proteopolymersome size and morphology are optimized through increasing polymer dissolution and reducing detergent removal rate.^102^ The successful incorporation of AQP0 in PEO‐PB and PMOXA‐PDMS‐PMOXA is determined with electron microscopy (EM), and the water permeability of AQP0 determined using stopped flow light scattering measurements showed permeability of 189.7 ± 61.3 μm/s,^102^ which is high compared to the measured permeability of other reported polymersomes, such as 2.5 μm/s for poly(ethyl ethylene)‐poly(ethylene oxide) (PEE‐PEO).^130^ This could be due to smaller hydrophobic repeat units in the PEO‐PB polymer compared to other polymer‐based polymersomes.^102^

Apart from MP studies, AQP incorporated polymersomes also can be applied in industrial water purification processes. For instance, AQPZ reconstituted PMOXA‐PDMS‐PMOXA proteopolymersomes are covalently immobilized onto the surface of a porous ultrafiltration cellulose acetate membrane, followed by in situ surface imprinting polymerization to generate a thin imprinted polymer layer.^101^ Forward osmosis and nanofiltration functionality were also tested and determined that AQPZ imprinted membrane had salt rejections above 50% and has a membrane selectivity of water to salt, demonstrating AQPZ facilitated water transport and salt rejection.^131^

#### 4.1.3 FhuA

Polymersomes have also been used to study transmembrane protein ferric hydroxamate uptake protein component A (FhuA), which is one of the largest β‐barrel channel proteins. In *E.coli*, FhuA mediates the active transport of ferrichrome‐bound iron and it also acts as the receptor for bacteriophages. Truncated variants of FhuA (FhuA Δ1–129 and FhuA Δ1–160) has been reconstituted in PMOXA‐PDMS‐PMOXA polymersomes using cell‐based reconstitution, and the activity of FhuA has been determined through monitoring the passage of sulforhodamine dye into polymersomes,^27^ or release of calcein dye out of the polymersomes via fluorescence spectroscopy.^103^ The direction of FhuA Δ1–160 insertion has been determined through measuring endodermic changes using isothermal titration calorimetry (ITC) in PMOXA‐PDMS‐PMOXA polymersomes.^132^ In a separate study, FhuA Δ1‐159 has been reconstituted into thick PIB‐PEG‐PIB polymersome membranes.^64^ To overcome the problem of hydrophobic mismatch that is seen during insertion of FhuA Δ1‐159, the length of MP can be matched to the thickness of the polymersome by doubling the last five amino acids of each of the 22 β‐sheets before the more regular periplasmatic β‐turns, which can lead to an 1 nm increase to become extended FhuA Δ1‐159 (FhuA Δ1‐159 Ext).^64^ The secondary protein structure of reconstituted FhuA Δ1‐159 Ext is determined through CD spectroscopy, which shows β‐barrel folding, indicative of correct folding. The functional activity of FhuA Δ1‐159 Ext is proven via kinetic analysis of 3,3′,5,5′‐tetramethylbenzidine (TMB) uptake by encapsulated HRP.^64^

#### 4.1.4 Ion channels

Ion channels, such as gramicidin A (gA),^104^ ionomycin,^105^ and KcsA^59^ have also been studied in polymersomes. Ion channel gA, which allows for the transport of protons and monovalent ions, is reconstituted in a series of PMOXA–PDMS–PMOXA polymersomes with membrane thickness ranging from 9.2 to 16.2 nm, where membranes thicker than 12.1 nm did not result in successful reconstitution of gA protein, potentially due to hydrophobic mismatch of the protein to polymersome membrane.^104^ The functionality of gA is investigated through encapsulation of pyranine, a pH‐sensitive dye in polymersomes, where quenching of fluorescence intensity indicates gA activity due to transport of protons into the polymersomes. Other methods such as monitoring for fluorescence changes of the Asante NaTRIUM Green‐2 (ANG‐2) dye that is specific for Na^+^ transport and Asante Potassium Green‐2 (APG‐2) dye that is specific for K^+^ transport have also been used to determine gA functionality (Figure 4d).^104^ Ionomycin, which allows for transport of Ca^2+^ ions, has been incorporated in PMOXA–PDMS–PMOXA‐based polymersomes or polymeric GUVs via film rehydration, and its transport functionality is studied through analyzing fluorescent increases in the calcium sensitive Asante Calcium Green (ACG) dye due to influx of Ca^2+^ ions into the polymersome.^105^ In addition, the permeability of ionomycin can be determined with stopped flow apparatus.^105^ KcsA, which allows for transport of K^+^ ions, has also been studied in PMOXA‐PDMS‐PMOXA polymersomes. However, the incorporation efficiency of the KcsA is only 5%, potentially due to the long drying process during electroformation, which results in aggregation and eventual degradation of the KcsA channel. KcsA insertion is confirmed with measuring the free lateral diffusion inside the polymer membrane with z‐scan FCS, where an increase in diffusion rate indicates incorrect incorporation due to protein aggregation.^59^

#### 4.1.5 Maltoporin/LamB

Maltoporin or LamB is a trimeric channel in the outer cell wall of Gram‐negative bacteria that specifically transport maltose and maltodextrins and also serves as a receptor for phage λ. LamB was reconstituted into PMOXA–PDMS–PMOXA polymersomes through mixing the LamB and vesicles solution together to mimic and analyze the mechanisms of phage genome transfer into bacteria through phage binding to trigger release of DNA into the polymersome.^28^ LamB functionality is determined through monitoring the change in Oxazole Yellow (YO‐PRO‐1) fluorescently labeled DNA released into the vesicle before and after phage addition. The addition of phage results in a steep increase in the fluorescence intensity, indicating that the protein is functional in inducing the injection of viral DNA.^28^ This successful reconstitution of LamB in polymersomes can serve as polymeric nanocontainer that is able to translocate DNA across a synthetic membrane, which can potentially be applied in gene delivery and therapeutic applications.

#### 4.1.6 TsX

TsX is nucleoside‐specific channel‐forming outer membrane porin that allows the specific transport of nucleosides and nucleotides. TsX has been reconstituted into PMOXA‐PDMS‐PMOXA polymersomes. To determine its nucleoside specific activity, the transport of prodrug 2‐fluoroadenosine into the polymersomes via TsX was monitored via its hydrolysis to 2‐fluoroadenine^106^ with a reducing sugar assay. TsX reconstituted proteopolymersomes are also used to deliver thymidine phosphorylase (TP) as an enzyme therapy strategy for mitochondrial neurogastrointestinal encephalomyopathy, where TsX functions as a channel to allow for the transport of enzyme substrate thymidine and product thymine through the polymersome. The TP enzyme activity can be determined through monitoring for thymine formation through determining the difference in absorption between thymidine substrate and thymine product at 290 nm.^133^

#### 4.1.7 MscL (hybrid vesicles)

MscL is a large‐conductance mechanosensitive ion channel found in prokaryotic and eukaryotic cell membranes and play an important role in rapidly regulating turgor pressure around the cell in response to increased membrane tension. Hybrid vesicles consisting of DOPC with varying concentrations of PEO‐PBD diblock copolymer are used to reconstitute and study the folding of α‐helical MscL.^51^ MscL protein is incorporated into the hybrid membrane via cell‐free expression using a construct of MscL tagged with monomeric enhanced green fluorescent protein (mEGFP) at the C‐terminus as well as a translation system. Proper folding of MscL results in an increase of GFP fluorescence intensity. The functional activity of MscL incorporation is investigated through a calcein dye release through measuring the amount of calcein release from the vesicle via fluorescence spectroscopy.^51^

The ability to add pores or synthetic channels to polymersomes could lead to novel membrane composites with unique selectivity and permeability. For instance, α‐hemolysin, involved in pore formation, has been inserted into PBD‐PEO polymersomes using cell‐free co‐translational incorporation approach, which increased permeability to encapsulated calcein dye.^52^ In addition to porins, synthetic pores self‐assembled from either a dendritic dipeptide or a dendritic ester have also been successfully synthesized into stable helical pores in PEO‐PBD polymersomes to enhance polymersomes permeability.^134^ Similarly, synthetic porins made from carbon nanotubes have also been incorporated in PBD‐PEO copolymer‐based polymersomes.^135^ Other functional modifications to polymeric membrane include incorporation of multiple channel proteins such as AlkL, OmpW, OprG and TodX, PhoE and FocA in PMOXA‐PDMS‐PMOXA polymersomes, where the combination of TodX and PhoE led to the most significant improvement in mass transfer compared to polymersomes without MPs.^136^ This study primary focuses on improving mass transfer of polymersomes and not biophysical characterization of the reconstituted MPs. Other applications of channel proteins reconstituted polymersomes include being nanoreactors, where the channel proteins allow for selective permeation of water, nucleotides, and molecules into polymersomes to facilitate enzymatic reactions.^96^, ^105^, ^136^

### 4.2 Protein complexes for active transport

#### 4.2.1 NADH:ubiquinone oxidoreductase (Complex I)

**FIGURE 5:**
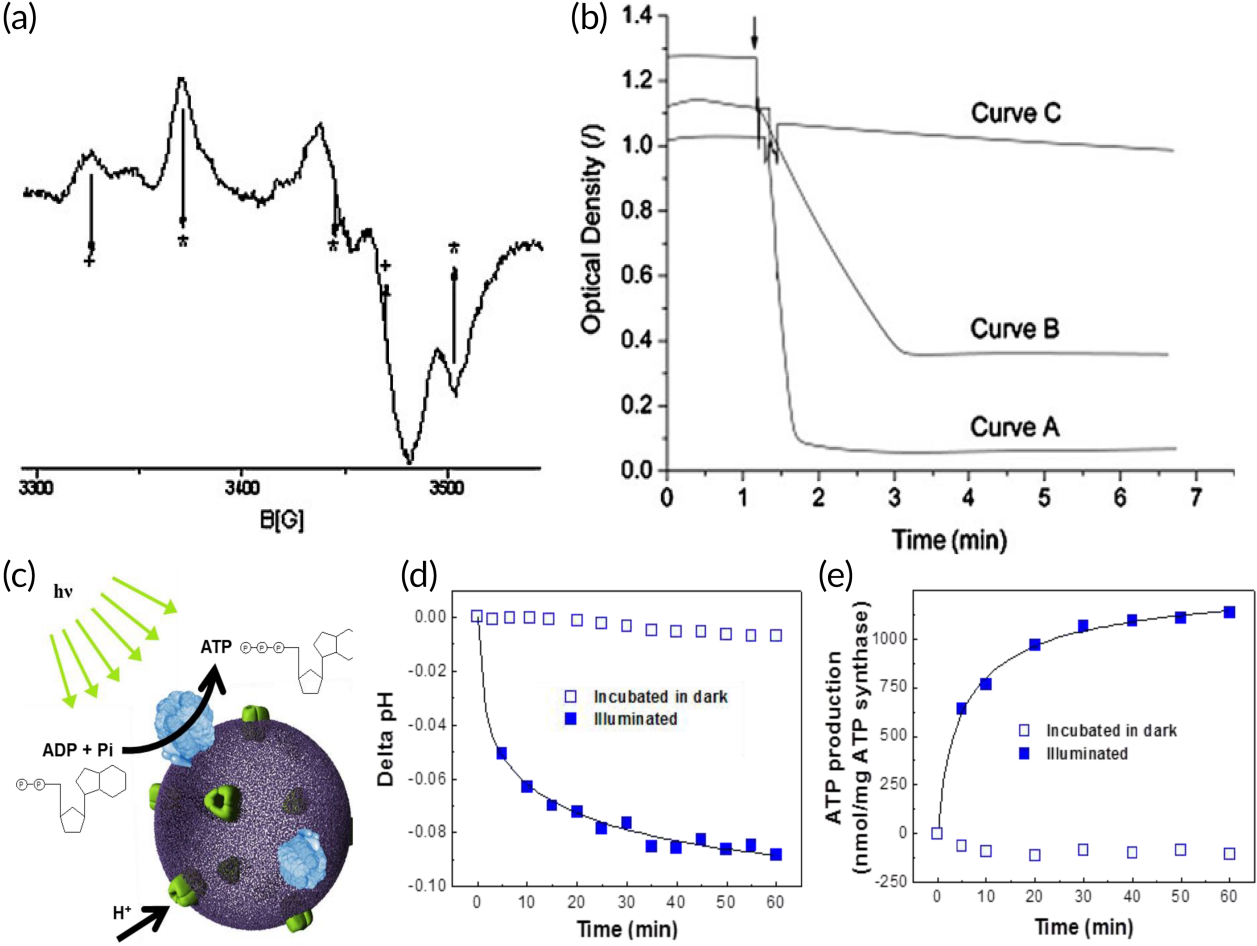
Structural and functional studies of membrane protein complexes with active transport in polymersomes. (a) Electron paramagnetic resonance (EPR) spectrum of NADH:ubiquinone oxidoreductase (Complex I) reconstituted in polymersomes. The EPR spectrum of Complex I in polymersome is similar to that of native Complex I solubilized in surfactants, indicating that chain of electron transfer was not affected by the reconstitution process. The five arrows with + and * indicate the signature of EPR spectrum specific to native Complex I.^137^ (b) Measurement of NADH/ferricyanide oxidoreductase activity to determine the preferential orientation of Complex I in polymersomes. The activity of native Complex I solubilized in surfactants (Curve A) is preserved after incorporation in the polymer membrane (Curve B), and Curve C indicates no activity from empty polymersomes. The reduction of activity in proteopolymersomes is due to reduced fraction of incorporated protein, as well as partially unoriented Complex I. *Source*: Figure 4a,b is reproduced with permission from reference 90, Copyright 2010, John Wiley and Sons. (c) Schematic representation of an ATP‐producing polymersome based on bacteriorhodopsin (BR)‐ATP synthase coupling system. (d) Intravesicular pH change with respect to light illumination as a measure of proton pumping activity of BR reconstituted polymersomes. (e) Photosynthetic ATP production in the BR‐ATP synthase reconstituted polymersomes under illuminated condition, which is coupled with proton pumping activity of BR. *Source*: Figure 4c–e is reproduced with permission from reference 61, Copyright 2005, American Chemical Society, as well as from reference 138, Copyright 2013, MDPI

Amphiphilic block copolymer PMOXA–PDMS–PMOXA was used to study the electron‐transfer activity of bacterial respiratory enzyme complex NADH:ubiquinone oxidoreductase (Complex I).^90^ Complex I couples the transfer of electrons from NADH to ubiquinone performed by a series of redox centers with a translocation of protons across the membrane. EPR, a well‐known technique to detect free radicals, was used to detect the presence of radical anions of the electron acceptors, which accounts for the in situ activity of Complex I in proteopolymersomes (Figure 5a).^90^, ^137^ NADH/ferricyanide oxidoreductase activity assay proved that a high fraction of Complex I was inserted with desired orientation, favoring electron transfer from the vesicles into their membranes (Figure 5b).^90^ Furthermore, ubiquinone 2 (CoQ2), known to be involved in the natural mechanism of energy conversion as an electron acceptor, was used to indicate the amount of electron transfer from the vesicles into their membranes. The addition of NADH to the proteopolymersome solution generated an EPR spectrum of CoQ2 with a significantly higher intensity, indicating the incorporation of more reduced forms of CoQ2 in the proteopolymersomes, which proves that Complex I mediate the electron transfers when reconstituted in the polymer membrane.^90^

#### 4.2.2 ATP synthase and bacteriorhodopsin (BR)

ATP synthase is composed of two domains, the membrane integrated F_0_ and the soluble F_1_. Coupling activity between the F_0_ and F_1_ complexes drives proton movement toward the F_1_ side of the membrane, resulting in ATP synthesis (Figure 5c).^61^, ^138^ The rotating activity of F_o_F_1_‐ATPase in the amphiphilic triblock copolymer PEtOz−PDMS−PEtOz can be maintained to synthesize ATP, using the photoinduced proton gradient generated from BR activity.^61^, ^62^, ^107^ This is the first successful biosynthesis through coupled reactions between reconstituted transmembrane proteins in a single proteopolymersome, and the first to demonstrate motor functionality in a polymer membrane. The production of photoinduced electrochemical proton gradients from both BR and BR‐ATP synthase reconstituted proteopolymersomes can be measured by the addition of pyranine inside the proteopolymersomes. The relative fluorescence intensity ratio at 456 nm and 402 nm indicates the H^+^ ion concentration, and hence the internal pH in proteopolymersomes can be quantified (Figure 5d).^61^, ^138^ A bioluminescence assay using luciferin and luciferase is used to quantify ATP production, since luciferase catalyzes the oxidation of luciferin by consuming ATP and shows that ATP production increases significantly with increasing light incubation, indicating functional reconstitution of ATP synthase (Figure 5e).^61^, ^62^, ^107^, ^138^ However, proteopolymersome‐based studies of F_o_F_1_‐ATPase are limited by difficulties involved in the reconstitution process, such as low membrane permeability due to its synthetic nature and material inhomogeneity, thereby preventing continuous substrate and products transport across the channel protein and reduction in enzymatic reactions. Furthermore, some reconstitution conditions can be harsh to the F_o_F_1_‐ATPase, which is made up of multiple subunits that can be easily disrupted. Therefore, there is a need for better optimized membranes such as hybrid vesicles formed by the blends of lipids and block copolymers that can result in better reconstitution of such MP complexes.^139^

#### 4.2.3 Proton pump—proteorhodopsin

Purified light‐activated photo pump proteorhodopsin (PR) can be reconstituted in polymersomes formed from PEG‐PDPA‐PSS.^70^ PR has a distinct polarity where the intracellular side has a slight positive charge, which is further increased through engineering a decahistidine‐tag at this side. On the other hand, the extracellular side bears a slightly negative charge. As a result, incorporation of PR into the polymersome allowed for its directed insertion where the PR would orientate with the negatively charged PSS group. This functionality of PR is confirmed by a light‐dependent pH change of the proteopolymersome solution, indicating the intended orientation.^70^ In another study, PR is reconstituted in polystyrene‐*b*‐poly(4‐vinyl‐*N*‐methylpyridine iodide)_2_ (PS‐P4MVP_2_) polymersomes via spontaneous reconstitution at pH 7.4.^108^ The membrane bilayer thickness is around 3.4–4.4 nm depending on increasing PS chain length, while the length of PR is less than 3.5 nm, indicating that hydrophobic mismatch may occur during reconstitution.^108^ However, the results show successful PR reconstitution, suggesting that the polymer membrane is conformationally active to match the hydrophobic domain of PR. The reconstitution and packing of PR in these proteopolymersomes are investigated with SAXS, revealing a two‐dimensional hexagonally packed PR lattice in individual proteopolymersome membrane bilayers, consistent with previously conducted orientation studies. The secondary structure and structural stability of PR was further confirmed using Raman and solid‐state NMR (ssNMR) spectroscopy through labeling with^13^C and^15^N radioisotopes.^108^ Time‐resolved visible spectroscopy through flash‐photolysis was used to determine PR functionality through monitoring whether it maintained key photocycle steps and turnover kinetics, where they showed that the PR reconstituted in proteopolymersomes retained the presence of M intermediate at 420 nm, absence of strong signals from the 13‐cis‐dark state at 600 nm, and relatively fast photocycle turnover kinetics.^108^

#### 4.2.4 Proton pump—cytochrome bo3 (hybrid vesicles)

The MP cytochrome bo3 (Cyt‐bo3), a redox‐reaction driven proton pump that couples oxygen reduction to proton transport, has been studied in hybrid lipid vesicles made from diblock copolymer PBD‐PEO and 1‐palmitoyl‐2‐oleoyl‐sn‐glycero‐3‐phosphocholine (POPC) phospholipid, with varying percentages.^89^ Hybrid vesicles are used because they can combine both the higher stability of polymer components and the more annular and biocompatible lipid bilayer. The hybrid vesicle is formed via optimization of the reconstitution techniques, where extrusion of the hybrid vesicles, followed by gradual destabilization of the vesicles by small amounts of detergents, and eventual incorporation of the MP yielded spherical vesicles with size between 75 and 116 nm, confirmed with DLS and TEM.^89^ To determine the optimal ratio between POPC and PBd_22_‐PEO_14_ that enables the highest Cyt‐bo3 activity in the hybrid vesicles, the initial rates of decylubiquinone oxidation are measured via absorbance reading at 275 nm, where an equimolar ratio between POPC and PBD‐PEO yields the best hybrid vesicle with Cyt‐bo3 having high initial activity and slow loss in activity.^89^ Comparatively, Cyt‐bo3 is not functionally reconstituted in PBD‐PEO only based polymersomes, due to the poor biocompatibility of its membrane, indicating the need for hybrid vesicles that combines POPC liposomes biocompatibility to high stability of the PBD‐PEO polymersomes.^89^ In a similar study with Cyt‐bo3 reconstitution in hybrid vesicles, the authors further investigated the hybrid membrane characteristics and showed that these membranes have less permeability than lipid bilayers, and 50 mol% PBD‐PEO hybrid vesicles have high initial reconstituted activity and retain around 20% of initial activity after 500 days.^140^ Cyt‐bo3 has also been reconstituted in PDMS‐*g*‐PEO with and without phosphatidylcholine (PC) and showed that it had the highest activity in hybrid vesicles, as measured by the level of oxygen reduction, while the activity in either polymersomes or liposomes was about the same.^141^

#### 4.2.5 NaAtm1 and human P‐glycoprotein (hybrid vesicles)

ATP binding cassette (ABC) proteins including *Novosphingobium aromaticivorans* Atm1 protein, which mediates the active efflux of toxic metals complexed to glutathione, and human P‐glycoprotein (Pgp), which transports hydrophobic drugs, have been reconstituted and studied separately in hybrid vesicles consisting of both phospholipids and PBD‐PEO.^30^ Reconstitution of either human Pgp or Atm1 protein is confirmed by density gradient centrifugation, as well as low passive permeability to a fluorescent probe (calcein acetomethoxyl‐ester) (C‐AM). Functional reconstitution of Atm1 or Pgp proteins is determined by ATPase functional assay which measures the liberation of inorganic phosphate.^30^

Besides the examples on Cyt‐bo3 proton‐pumping oxygen reductase and ABC transporters, transmembrane protein complexes have a primary application of ATP production, which is coupled to active transport of protons under light stimulation.^142^ Research has focused on optimizing artificial photosynthetic systems for ATP production to advance toward engineering of artificial cells. A limitation of the current approach lies in ATP being produced outside proteopolymersomes or proteoliposomes, which does not allow for more quantitative mechanistic studies such as mimicking in‐cell biochemical reactions. An improvement to this has been reported in a study using liposome GUVs to produce ATP where multilayer vesicles were formed like the structure of plant cells and ATP was harvested in the inner membranes to drive actin polymerization and carbon fixation continuously.^143^ More MPs capable of energy harvesting could be reconstituted in polymersomes^94^ to study their energy production capability as well as expand the research on artificial cells that can perform generation and consumption of energy all within themselves.^144^, ^145^

## 5 MEMBRANE RECEPTORS

Membrane receptors are specialized protein molecules attached to or integrated into the cell membrane. Membrane receptors play important roles such as facilitating communication between the cell and the extracellular environment through interaction with specific ligands including hormones and neurotransmitters.^146^ Membrane receptors have been studied in liposomes; however, the incorporated proteins are unstable and hinder the measurements of receptor functions.^147^ Hence, receptor‐based proteopolymersome systems have been engineered with reconstitution of receptors that are responsible for signal transduction (G‐protein‐coupled receptors, GPCRs), cell–cell communication (Cldn2), immune response (major histocompatibility complex I, MHC‐I) and cell adhesion (peptide anchors) (Table 1).

### 5.1 GPCRs (DRD2, CXCR4, and GLP‐1R)

**FIGURE 6:**
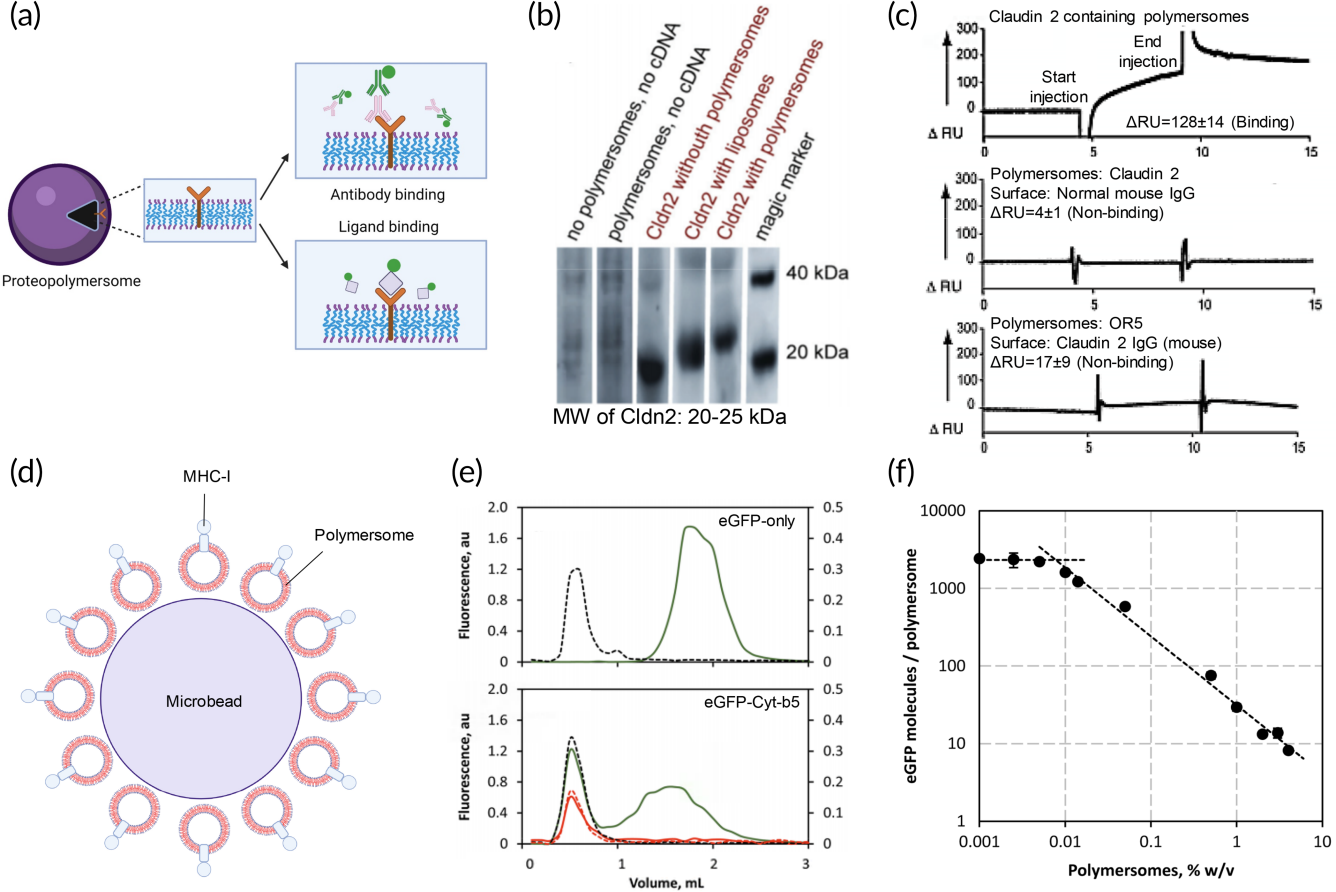
Characterization of membrane receptor‐based proteopolymersomes and their applications. (a) The structure–function relationship of membrane receptors is characterized by binding of conformational antibodies and native ligands to illustrate the proper folding and functions of the receptors, respectively. Schematic was created with BioRender.com. *Source*: Reproduced with permission from reference 53, Copyright 2012, John Wiley and Sons.(b) Western blot of in vitro expressed claudin‐2 (Cldn2) in the absence or presence of polymersomes or liposomes. (c) SPR measurements showing the binding of Cldn2 reconstituted proteopolymersomes to anti‐Cldn2 functionalized surface but not normal mouse IgG surface. There is also no significant binding between empty polymersomes without Cldn‐2 expression and the anti‐Cldn2 surface.*Source*: Figure 5b,c is reproduced with permission from reference 52, Copyright 2011, Springer Nature.(d) Engineering of an artificial antigen‐presenting cell by cell‐free in vitro synthesis and incorporation of MHC‐I into polymersomes (red vesicles) and the attachment of MHC‐I proteopolymersomes onto the 3D surface of microbead as a support (purple), forming MHC‐I proteopolymersome‐beads.^111^ (e) Size‐exclusion chromatography (SEC) characterization of eGFP (top) and eGFP‐fused transmembrane domain of the rabbit cytochrome b5 (Cyt‐b5) (bottom) proteopolymersomes. Black dashes represent quantification of polymersomes by measuring light absorbance at 350 nm. Green and red solid lines show the presence of eGFP characterized by fluorescence signal (green, made fresh; red, after 6 weeks of storage). (f) There is an inversely proportional dependency of the immobilized eGFP‐Cyt‐b5 molecules per polymersome with increasing polymersome concentration. The polymersome surface area becomes limiting below 0.05% w/v. *Source*: Figure 5e,f is reproduced with permission from reference 113, Copyright 2016, Springer Nature. Schematics were created with BioRender.com.

GPCRs represent the largest class of MPs in the human genome and play a key role in mediating most of our physiological responses to neurotransmitters, hormones, and external stimuli. Hence, they are potential therapeutic targets for a broad spectrum of diseases and the study of their structure–function relationship is important.^148^ Several proteopolymersome systems with GPCRs incorporation have been generated through cell‐free synthesis, including the reconstitution of dopamine receptor D2 (DRD2),^53^ chemokine C‐X‐C receptor 4 (CXCR4)^109^ and glucagon‐like peptide‐1 receptor (GLP‐1R) into polymersomes formed by PMOXA‐PDMS‐PMOXA or PBD‐PEO block copolymers.^110^ In these proteopolymersomes, successful GPCR insertion is characterized by flow cytometry, SEC, and Western blots. The physiologically correct folding and orientation of reconstituted GPCR is confirmed by binding of respective conformational specific antibodies and native or synthetic ligands (Figure 6a),^53^ as characterized by surface plasmon resonance (SPR), flow cytometry, I‐125 radioactive ligand binding or fluorescence‐based assays, with non‐GPCR proteopolymersomes or polymersomes without MP incorporation used as controls which showed no binding.^53^, ^109^, ^110^

In the individual system, the binding of dansyl‐labeled dopamine to DRD2 proteopolymersome illustrates a half‐maximal effective concentration (EC_50_) of 30 μM, which is much higher than the known EC_50_ of its native ligand dopamine in the nanomolar range.^149^ While it is not discussed, this discrepancy can be due to the low amount of protein incorporation at only 25%, the presence of dansyl label leading to steric hindrance, and potential protein misfolding due to cell‐free synthesis that resulted in reduced ligand binding capacity. In the CXCR4 system, comparable dissociation constants of native ligand SDF‐1α in CXCR4 proteopolymersome (8.4 nM) and native membrane (1.4 nM) are identified. It was suggested that the lower affinity of the ligand for proteopolymersome could be due to the absence of G proteins in the synthetic system, which may affect CXCR4 conformation and alter ligand binding affinity.^150^ In the GLP‐1R study, the binding affinity (K_d_) of N‐terminal extracellular domain specific antibody is 18.6 nM, which indicates that some GLP‐1R assumed a correct orientation due to the accessibility to the N‐terminal domain. However, there is also some binding of the 1D4 antibody to the C‐terminal C9 tag, suggesting the presence of reversely incorporated GLP‐1R in the proteopolymersomes.^110^ In addition, the low SPR response units during antibody binding show the presence of a low percentage of GLP‐1R incorporation into polymersomes. To promote folding of GLP‐1R for enhanced functionality, Fos‐choline 14 (Fos14) detergent is introduced, which functions as a chemical chaperone. Fos14 assists the folding of GLP‐1R and mediates a more stable incorporation of GLP‐1R into the polymersomes.^110^ Radioligand competition binding assay between^125^I‐labeled GLP‐1 as tracer and native peptide ligand exendin‐4 confirms the functionality of these Fos14‐assisted GLP‐1R proteopolymersomes. The K_d_ of GLP‐1R proteopolymersomes (54.3 nM) determined is similar to that of GLP‐1R in native membrane (37.8 nM).^110^

### 5.2 Claudin‐2

Claudin‐2 (Cldn2) is a transmembrane receptor that promotes cell–cell adhesion by forming homodimer with another molecule in neighboring cell.^151^ Cldn2 is a component of the tight junction and forms cation‐selective and water permeable paracellular channel.^151^ It also acts as a signal modulator and integrator that affects cell proliferation and migration, which may be relevant in both cancer biology and tissue regeneration.^151^ Cldn2 is inserted into PBD‐PEO polymersomes using a cell‐free in vitro synthesis method and characterized for reconstitution using SEM and Western blots (Figure 6b).^52^ Staphylococcal α‐hemolysin, which is a pore‐forming MP, is used as a positive control through dye leakage assay to demonstrate spontaneous MP insertion into PBD‐PEO polymersome. Cldn2 proteopolymersome is also characterized by monitoring the binding of specific antibodies against Cldn2 using SPR. SPR measurements indicate that there is binding between Cldn2 proteopolymersomes and the immobilized anti‐Cldn2 IgG (ΔRU of 128 ± 14) but not with normal mouse IgG (ΔRU of 4 ± 1) functionalized surface (Figure 6c).^52^ There is no significant binding between polymersomes without Cldn‐2 expression and the anti‐Cldn2 IgG functionalized surface (ΔRU of 17 ± 9). Cldn2 has also been reconstituted into liposomes for direct comparison between the functionality of incorporated protein in both types of nano‐vesicles. The increased binding to anti‐Cldn2 by Cldn2 proteopolymersomes as compared to Cldn2 proteoliposomes not only indicates the correct folding and orientation of reconstituted Cldn2 but also the enhanced stability of protein insertion into polymersomes than liposomes for MP studies.^52^

### 5.3 MHC‐I

To induce immune‐modulatory response, it is essential for MHC‐I proteins to be expressed on the extracellular‐side of antigen‐presenting cells (APCs) for molecular recognition of pathogens by T cells. Artificial APCs, which can behave as polymer‐based synthetic immunological synapses, are often used to enhance MHC‐I antigen presentation.^152^ A new type of artificial APC is developed using cell free in vitro synthesis method of incorporation of MHC‐I molecule H‐2Kb preloaded with chicken ovalbumin (OVA) into the bilayer membranes of ABA‐RBOE‐PS‐SA nano‐vesicle beads that are made from self‐assembly of block copolymers (Figure 6d).^111^ After confirming the structure and function of the incorporated MHC‐I, the MHC‐I H‐2Kb‐OVA proteopolymersomes serve as artificial APCs to promote antigen recognition and immunological synapse formation in CD8^+^ T cells isolated from OT‐I transgenic mice and induced T‐cell activation.^111^ The engineered MHC‐I proteopolymersome represents a promising platform for studying and quantifying the spatio‐functional interactions between artificial APC and T‐cell and hence can have further applications such as HTS of T‐cell regulating compounds. In another study, pH‐responsive nanoparticles composed of triblock copolymers ([BMA‐*co*‐DEAEMA]‐b‐[DMA‐*co*‐PDSMA] polymers) doped with pyridyl disulfide functionalized monomer (PDSMA) for antigen conjugation are incorporated with MHC‐I, for use as artificial APCs.^153^ Although different from MHC‐I proteopolymersomes, the MHC‐I conjugated nanoparticles are able to enhance MHC‐I antigen uptake in dendritic cells, consistent with that observed in MHC‐I proteopolymersomes.^153^

### 5.4 Peptide anchors (CecA, Cyt‐b5, Vam3p, lysis protein L)

Amphiphilic peptides have been used as anchors to decorate polymersome for additional surface functionality including anti‐microbial activity as well as for membrane surface anchoring of water soluble proteins.^154^, ^155^ An example is the reconstitution of a fusion protein (CecA‐eGFP) based on the antibacterial peptide Cecropin A (CecA) and the enhanced green fluorescent protein (eGFP) into polymersomes formed by triblock copolymer polyisobutylene‐polyethylene glycol‐polyisobutylene (PIB–PEG–PIB).^112^ Successful reconstitution of CecA into polymersomes is characterized by the folding of a random coil into α‐helix in presence of polymersomes detected by CD and the co‐localization of CecA and polymersomes as shown through SEC and tryptophan fluorescence measurements.^112^ A follow‐up study has shown a similar reconstitution of natural peptide anchors including eGFP fused transmembrane domains of cytochrome b5 (Cyt‐b5), viral lysis protein L of the bacteriophage MS2, and yeast syntaxin VAM3 (Vam3p) with CecA‐eGFP as a positive control.^113^ The presence of natural peptide anchors allows the tethering of water‐soluble protein or enzyme to membranes. These natural peptides are reconstituted into PMOXA–PDMS–PMOXA polymersomes. The display of eGFP on the surface of polymersomes illustrates the proper insertion of the peptide anchors into the polymeric membranes and co‐localization of these peptides and polymersomes is shown through SEC (Figure 6e).^113^ The study also shows an inversely proportional dependency of the immobilized eGFP‐Cyt‐b5 molecules per polymersome with increasing polymersome concentration where the polymersome surface area becomes limiting below 0.05% w/v (Figure 6f).^113^ Importantly, these peptide anchors do not form pores or disintegrate the membranes, illustrating their potential to anchor water soluble proteins on membrane surface.^113^, ^154^

While the above membrane receptor studies are conducted in proteopolymersomes, there are other receptor‐based studies performed in proteoliposomes as well as in lipid and polymer bilayers.^156^, ^157^, ^158^, ^159^, ^160^, ^161^, ^162^, ^163^ Some of these important MP complexes, such as β‐site amyloid precursor protein (APP) cleaving enzyme 1 (BACE1) and γ‐secretase,^156^, ^157^, ^158^, ^159^, ^160^ may be further studied in polymersome for structural and functional comparison in different nano‐vesicles. The techniques used in these liposome‐related MP studies, including FCS, fluorescence recovery after photobleaching (FRAP), single molecule tracking (SMT), total internal reflection fluorescence spectroscopy (TIRFS), fluorescence resonance energy transfer (FRET) and continuous‐wave EPR (CW‐EPR), could also be applied to the characterization of receptor–ligand interactions and changes in MP conformations and oligomeric states in proteopolymersomes.^161^, ^162^, ^163^

## 6 FACTORS AFFECTING MP STUDIES IN PROTEOPOLYMERSOMES

A good cell membrane mimetic should be morphologically similar to the biological bilayer membrane, equivalent thickness in a liquid crystalline phase, without any change in the membrane fluidity, which may affect the equilibrium distribution of the different MPs.^164^ There are several factors that affect the folding, function, and dynamic equilibrium of MPs in proteopolymersomes. We will discuss three key groups of factors below, including membrane composition, MP expression and reconstitution system, and protein states (Table 2).

**TABLE 2:** Factors affecting membrane protein (MP) reconstitution and quality of structural and functional characterization

Optimization parameters	Factors affecting MP reconstitution	Effects on MP structural and functional characterization	MPs	References
Membrane composition	Polymer composition/asymmetricity	Determine the physiologic orientation and function of the inserted MP; molecular weights for different polymer blocks can facilitate efficient protein encapsulation and stabilization	AQP0 PR	29, 70, 165
	Polymer flexibility/curvature	Determine the physiologic orientation and function of the inserted MP	AQP0 OmpF	29, 166, 167
	Polymer membrane thickness	Increased thickness increases MP conformational stability	Complex I	28, 90, 166, 168
Membrane protein expression and reconstitution system	Cell‐based protein purification and reconstitution	Advantages: Addition of detergents allows for ease of MP solubilization and can facilitate MP folding Disadvantages: Cytotoxicity, misfolding, and aggregation	OmpF Cldn‐2	169, 170, 171
	Cell‐free co‐translational incorporation	Advantages: No cytotoxicity, pure and homogenous protein formation, can be solubilized with milder detergents, less chance of protein misfolding Disadvantages: Lower final protein yields	OmpF Cldn‐2	169, 170, 171
	Hydrophobic mismatch	Decrease the equilibrium concentration or activity of MP	OmpF	29, 64, 167, 172, 173
	Detergent concentration	High concentrations may decrease MP activity	NaAtm1 Pgp FhuA OmpF	30, 101, 132
	Rate of detergent removal	Slow rate of removal results in smaller polymersomes; fast rate of removal results in larger polymersomes	AQP0	102
	Size of vesicles	Smaller vesicles are more compatible with biological membranes and increases MP stability; large vesicle interior volume lowers MP membrane concentration, and increases NMR structural study difficulty	Cldn‐2	52, 174, 175
	Preformed vesicles	Limit the number of MP that can be incorporated due to high energetic expenditure	AQP0	102
	Polymer/lipid‐to‐protein ratio	A low polymer/lipid to MP ratio increases the quantity of MPs available in polymersomes for study; a ratio of 1:1 has been shown to have high NMR sensitivity	MsCL Influenza M2 proton channel	176, 177
	Protein amount/concentration	High protein concentration increases MP reconstitution; saturated protein concentration decreases MP reconstitution	OmpF TsX	25, 26, 27, 28, 60, 96, 97, 102, 106
Protein states	Monomers/purple membrane	Determine the physiologic orientation and function of the inserted MP	BR/F_0_F_1_‐ATP synthase	107
	Environmental condition	Light illumination increases MP activity	PR	60, 61, 62, 107
	Immobilization of polymersomes on surfaces/free flowing polymersomes	MP activity decreases when polymersomes are immobilized	OmpF	96
	Correlation time of the MP–surfactant complex (PSC)/Tumbling rate	Determine the ability of protein structure to be resolved by NMR. Fast‐tumbling small PSCs (<100 kDa) can be studied by solution NMR; slow reorienting aggregates are more suitable to be characterized by solid‐state NMR	DAGK	178, 179

### 6.1 Membrane composition

**FIGURE 7:**
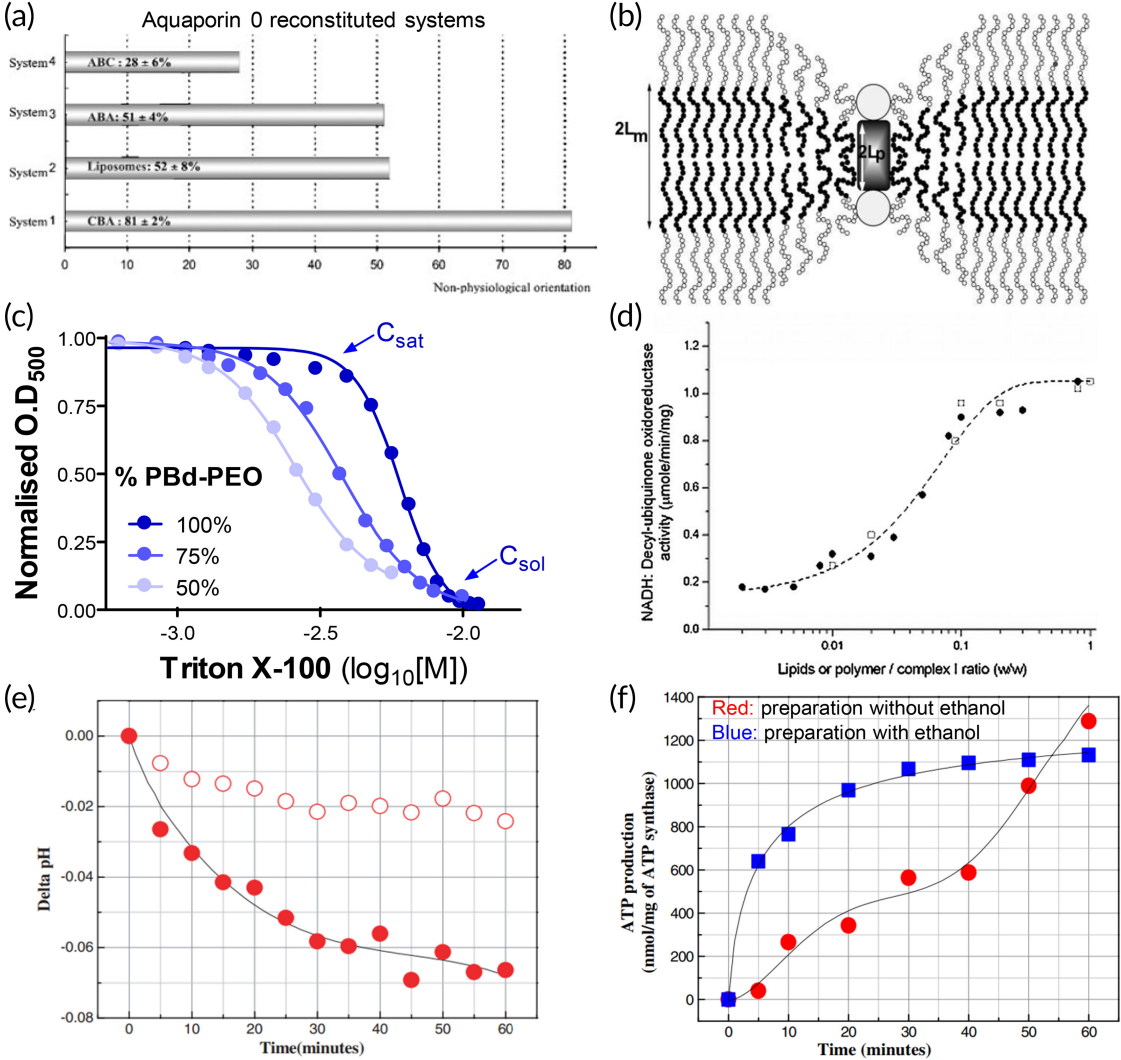
Factors affecting reconstitution efficiency and quality of membrane protein study. (a) Polymer asymmetricity affects orientation of aquaporin 0 insertion into polymersomes. *Source*: Reproduced with permission from reference 29, Copyright 2004, John Wiley and Sons. (b) Hydrophobic mismatch between the polymer and membrane protein near an inclusion in a polymeric bilayer. The chains in the unperturbed bilayer are highly stretched in order for much shorter proteins to match the thickness without undergoing significant compression when compared to the free chain radius of gyration. 2L_m_ is the thickness of a flat bilayer and 2L_p_ is the inclusion thickness. *Source*: Reproduced with permission from reference 166, Copyright 2003, Elsevier. (c) Higher detergent concentrations are required by vesicles made up by a higher proportion of block copolymers (PBd‐PEO) to reach saturation (C_sat_) and solubilization (C_sol_) points, indicating that there is an increase in stability with inclusion of block copolymers in vesicles. *Source*: Reproduced with permission from reference 30, Copyright 2020, MDPI. (d) Effect of increasing lipid/polymer to NADH:ubiquinone oxidoreductase (Complex I) concentration on decyl‐ubiquinone oxidoreductase activity. Increasing lipid/polymer to membrane protein ratio results in more functional activity. *Source*: Reproduced with permission from reference 90, Copyright 2010, John Wiley and Sons. (e) Preparation method controls proton vectoriality in bacteriorhodopsin (BP) proteopolymersomes. Incorporation with BR monomer in the absence of ethanol results in the light‐induced change in pH exhibiting negative values with increase in illumination time. Solid circles indicate illuminated condition, while hollow circles indicate dark‐incubated. (f) Protein states affect the functional activity of the reconstituted membrane proteins in polymersomes. BR‐ATP synthase exhibits an acceleration of ATP synthesis when polymersomes are prepared without ethanol (red). Preparation of polymersomes with ethanol (blue) shows an initial increase in ATP synthesis which decreases with time. *Source*: Figure 7e,f is reproduced with permission from reference 107, Copyright 2006, IOP Publishing

The asymmetricity of copolymers used in polymersome synthesis could result in different thickness and fluidity of the formed polymersomes, thereby affecting the orientation and functionality of the inserted MPs. A study with aquaporins with His‐tag shows that the percentages of nonphysiological orientation as characterized by the exposure of His‐tag to the external solution for lipids and ABA triblock copolymer were near 50%, which is in reasonable agreement with random insertion into the membranes. However, for ABC and CBA triblock polymers, there was a preferred physiologic orientation of 72% in the ABC system, and a nonphysiologic orientation of 81% in the CBA system (Figure 7a).^29^ In addition, designing triblock copolymers with different molecular weights for the A and C blocks can facilitate efficient protein encapsulation and stabilization. This can be done through having the longer end with a higher molecular weight segregating on the outside of the polymersome due to a larger radius of curvature with a differing volume fraction, and the smaller end segregating on the inside of the polymersome.^165^ The conformational freedom and flexibility of the polymers are key factors to promote MP incorporation without involving a loss of free energy. Hydrophobic mismatch between the polymer and MP affects protein structure and functionality (Figure 7b).^166^ For instance, in an OmpF reconstituted proteopolymersome, a thin 3 nm polymer bilayer matches with the protein length and results in functional protein without deformation of the polymer membrane. In contrast, a 6 nm polymer bilayer shows a strong negative mismatch, resulting in symmetric deformations in the upper and the lower leaflets, and could potentially lead to an expulsion of the MP.^167^ A membrane formed from amphiphilic block copolymers can withstand larger hydrophobic mismatches of more than 22% in the membrane thickness than lipid‐based membranes, which can typically only withstand mismatches of 2%–3%.^167^ Thus, increased flexibility of polymeric membranes can lead to a more successful biomolecule insertion. On the other hand, PR has been functionally reconstituted into highly stiff and stable glassy block copolymer membranes with the polystyrene hydrophobic block,^167^ indicating that the conformation and flexibility also depends on the type of MPs inserted.

Nano‐vesicles, in particular the SUVs, are more stable, have a smaller curvature than GUVs, and provide a local environment that is more similar to that of biological membranes for MPs. This property makes them more suitable for MP studies, particular for structural studies.^20^, ^43^, ^180^ In addition, a key advantage of these nano‐vesicles lies in the clearly defined compartments segregated by the copolymer layers, which creates a concentration gradient that allows the transport of solutes and hence the measurement of the activity of pore‐forming proteins across the membrane.^104^, ^123^, ^181^ Hence, controlling the size of the polymersomes is important in determining their applications and methods used to study MPs in proteopolymersomes.^182^ Polymersomes, although stable and come with many benefits, are not always favorable environments for MP reconstitution, and in some cases, modifications to the membrane environment are required to achieve the desired functions.^30^, ^183^ These issues motivate the modification of polymersome properties to enhance their bio‐functionality, such as blending block copolymers and phospholipids to create hybrid vesicles, with the goal of combining the best features of these two materials such as having the chemical versatility and robustness of polymersomes with the biocompatibility and biofunctionality of liposomes (Figure 7c).^30^, ^183^, ^184^

### 6.2 MP expression and reconstitution system

Cell‐free protein expression systems have been widely adopted to produce structurally intact mammalian MPs,^185^ and to overcome the limitations of the conventional protein production with use of *E. coli* or yeast.^116^, ^176^ Cell‐free protein production generates a large amount of properly folded and biologically active proteins to be mixed with sufficient copolymers for optimal reconstitution for extensive MP studies. While cell‐free approaches come with many advantages, the required enzymes supplemented in vitro which may be inferior to the quality control systems found in cells, and may contribute to making some misfolded and inactive MPs in the mixture that confounds quantifications of MP properties.^186^ Therefore, it is important to optimize the protein expression and reconstitution system to use for MP studies depending on the quantity and stability of the MPs required. Choice of detergents and organic solvents in reconstitution may affect the stability of MPs, and hence the efficiency of reconstitution for MP studies. The use of detergents is important in the extraction of large quantities of MPs which is required for techniques such as NMR to achieve a significant detection signal. However, high concentration of detergents used can also lead to formation of detergent micelles which can destabilize MPs. Consequently, careful control of detergent concentration needs to be done to increase MPs stability.^166^, ^176^, ^187^ Organic solvents are necessary for polymersomes solubilization and are used to mix with the MPs solution to facilitate protein reconstitution. However, the presence of organic solvents can denature MPs, hence decreasing their functional activity. Therefore, new methods such as polymer rehydration and droplet microfluidics, which eliminates the need for organic solvents, have been discovered for reconstitution to improve MPs quality and activity in multiple studies.^27^, ^43^, ^61^, ^62^, ^90^, ^107^ The lipid/polymer‐to‐protein concentration and ratio also affects MP activity.^188^ For instance, a ratio of 1:1 results in the highest NADH‐decylubiquinone oxireductase activity (Figure 7d).^90^

### 6.3 Protein states

The protein states of MPs used also affect its orientation of insertion in the polymersomes. This can be seen through the comparison between insertion of BR in monomeric state and BR in the form of purple membrane (PM).^61^, ^62^, ^107^ Using the BR monomer, results showed that light‐induced pH exhibited negative values with increasing illumination time (Figure 7e). This indicates that protons were being pumped inwards into the core of the polymersomes, which is opposite to the outward pumping with BR in purple membrane. This also shows that BR molecules are preferentially positioned with the C‐terminus facing outward and inward in the proteopolymersomes when reconstituted with BR monomer and BR in purple membrane respectively.^61^, ^62^, ^107^ Changes in protein states also affect the functional activity of MPs. In the case of proteopolymersomes of BR in PM state, BR‐ATP synthase exhibited an acceleration of ATP synthesis (Figure 7f).^107^ When the reconstituted BR is in the monomeric state, BR‐ATP synthase activity had an initial slow activity and increased progressively over the course of 30 min when the rate decreased.^107^

In a reconstituted proteopolymersome, changes in protein concentration used or amphiphile‐to‐MP ratio can affect proteopolymersomes morphologies, quality of protein crystals needed for MP structural study,^102^, ^176^ as well as MP orientation and activities,^52^, ^61^, ^62^, ^107^ and quantities available in proteopolymersomes for study. The use of excellent quality of protein crystals has also been shown to allow the elucidation of small molecule interactions with influenza M2 proton channels in lipid bilayers as well as the determination of the high‐resolution structures of their complexes.^177^ The correlation time of the protein‐surfactant complex (PSC) also affects the ability of MPs to be resolved by NMR, where the fast‐tumbling small PSCs below 100 kDa (e.g., diacylglycerol kinase [DAGK] in detergent micelles) can be studied by solution NMR,^178^ while slow reorienting aggregates are more open to ssNMR.^179^ Finally, there is a difference in the functionality of MPs inserted into free vesicles as compared to immobilized vesicles. It is observed that MPs in immobilized vesicles have 6.5 times lower activity than the free vesicles in solution.^189^ Two possible reasons can explain this phenomenon. First, it can be due to the presence of an unstirred aqueous layer at the polymer‐membrane solution interface leading to the formation of a diffusional barrier for otherwise rapidly permeating substrate.^189^ Second, the positioning of the nanoreactors toward the surface and the immobilization on the solid support may result in a reduced accessibility of the MPs.^96^

## 7 CELL MEMBRANE MIMETICS AS PLATFORMS FOR HTS IN DRUG DISCOVERY

**FIGURE 8:**
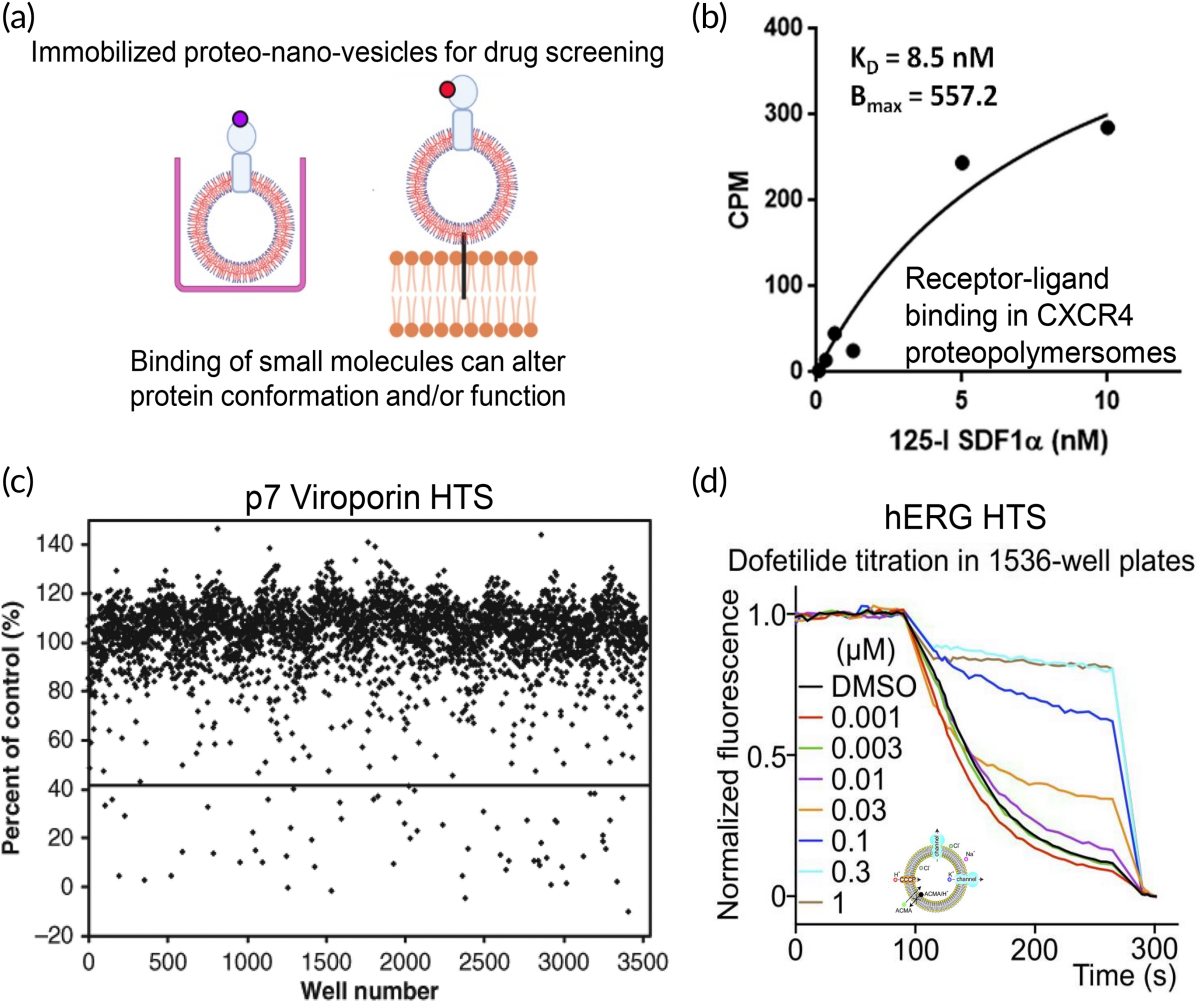
Proteopolymersomes and proteoliposomes as high‐throughput screening (HTS) platforms for drug discovery. (a) Immobilized proteopolymeromes or proteoliposomes, either through free‐flowing proteo‐nanovesicles residing in multiwell plates (left) or tethering of proteo‐nanovesicles to a bilayer support (right), can be used for HTS drug discovery. Binding of small molecules can alter membrane protein conformation and/or function. (b) Saturation binding of iodine‐125 (^125^I) radiolabeled SDF1α ligand to CXCR4 receptor incorporated proteopolymersomes as a proof‐of‐concept study that the proteopolymersomes can be utilized to screen for small molecule binders that modulate membrane receptor structures and functions. *Source*: Reproduced with permission from reference 109, Copyright 2014, PLOS. (c) HTS drug discovery of 3520 compounds using Hepatitis C Virus p7 Viroporin proteoliposomes and a fluorescence dye permeability assay. A 1.8% hit rate is observed. *Source*: Reproduced with permission from reference 194, Copyright 2011, SAGE. (d) A HTS platform in 1536‐well plates developed using hERG channel proteoliposomes and a fluorescence dye permeability assay. A positive control compound, dofetilide, is illustrated with an increase in dye permeability, indicating the ability of the HTS platform to be used to screen for compounds that modulate the hERG channel. *Source*: Reproduced with permission from reference 195, Copyright 2016, National Academy of Sciences, USA. Schematics were created with BioRender.com.

New in vitro tools and models that can directly monitor the structural and functional properties of MPs are increasingly needed to enable the identification of novel lead compounds that can guide preclinical drug developments. Currently, majority of the HTS campaigns in drug discovery make use of cell‐based biosensors and related secondary assays to identify small molecule modulators that target MPs. Although cell‐based platforms have multiple advantages including being more physiologically relevant, nonspecific targeting of drug compounds remains as a major limitation as drug compounds can interact with multiple proteins or targets in the cells. They also have the disadvantage of random insertion of gene of interest into the cell genome that can disrupt the expression of some endogenous proteins.^190^, ^191^, ^192^, ^193^ Hence, the characterization of the interactions between drug candidates and MPs using a cell‐free system to directly observe their functional modulation and structural perturbation in a high‐throughput setting can greatly facilitate the speed, specificity, and quality of drug discovery.^192^ MP inserted nano‐vesicles such as proteopolymersomes and proteoliposomes, either freely residing in microplates or immobilized onto a membrane bilayer, serve as excellent cell‐free HTS platforms (Figure 8a).^196^, ^197^

### 7.1 Structural and conformational screening

HTS of small molecules can be performed based on either modulating the protein–protein interactions (PPIs)^198^, ^199^ or perturbation of protein conformational dynamics, which directly corresponds to protein functions.^200^, ^201^ For example, multiple receptors form preligand dimers or undergo conformational changes upon ligand binding to exert their functions. The disruption of these interactions or alterations in their conformational states can lead to either inhibition or activation of the receptor.^202^ Biophysical techniques can be adopted to measure PPIs and protein conformational change for MP studies, such as using FRET^203^ or SPR^204^, ^205^, ^206^ in binding screens. While not yet established in proteopolymersome, the feasibility of this approach has been demonstrated through FRET measurement of the dimerization of transmembrane fibroblast growth factor receptor 3 (FGFR3) helix incorporated in proteoliposomes, where the determined thermodynamic parameters are directly relevant to the biological processes in cell membranes.^159^ This suggests that the compound induced modulation of MPs incorporated in proteoliposomes can be monitored through FRET changes, which directly reflect structural changes of MPs that correlate to their functions. In addition, high‐throughput binding screens can be conducted using SPR^204^, ^205^, ^206^ to identify small molecule inhibitors/activators that modulate PPIs. For example, SPR has been used to measure the binding of native ligands on Cldn2, DRD2, CXCR4, and GLP‐1R proteopolymersomes (Figure 8b).^52^, ^53^, ^109^ These platforms can also be optimized and adopted for HTS of active drug compounds that bind to these proteopolymersomes and alter the ligand binding capability to the reconstituted MPs, although no such study has been reported up to date. Nevertheless, studies have shown that it is feasible to detect compounds that alter structures and functions of the reconstituted MPs in proteoliposomes immobilized on SPR chips.^87^, ^197^ SPR measurements and screening have been done in immobilized lipid bilayers with reconstituted full‐length BACE1 and the interaction between the BACE1 and several inhibitors is confirmed by this SPR biosensor‐based assay.^207^

### 7.2 Functional screening

In MP functional screening, a direct method is to screen for compounds that modulate channel proteins through leakage assay of fluorescent dyes. While the use of proteopolymersome in functional screening of compounds targeting channel proteins has not yet been developed, this functional HTS strategy has been widely adopted in proteoliposomes; hence, a similar strategy can be potentially adopted.^20^ Examples include the development of novel cell‐free HTS methods for hepatitis C virus p7 viroporin^194^ and K^+^ channel proteins^195^ (Figure 8c,d). Specifically, a low‐throughput proteoliposome‐based fluorescent dye permeability assay was modified, optimized, and converted to a robust HTS assay to screen for compounds capable of interfering with p7 channel function (Figure 8c).^194^ To eliminate nonspecific hits, melittin channel‐forming peptide is used in a counter screen.^194^ Similarly, a proteoliposome flux assay using a fluorescent dye was applied in a HTS and the study identified new activators and inhibitors of four different K^+^ channels. GIRK2, TRAAK, Slo1 and hERG, all of which are important MPs to control ion homeostasis and cell signaling (Figure 8d).^195^ In another study, hERG channel is expressed through a cell‐free expression system and integrated into a biomimetic lipid bilayer platform. The properly folded and functional hERG channel is used for probing the channel and drug interactions through a fluorescence polarization assay and can be adopted for HTS to discover novel channel blockers.^208^ All these studies illustrate the potential of using proteopolymersome for similar functional screening studies.

**FIGURE 9:**
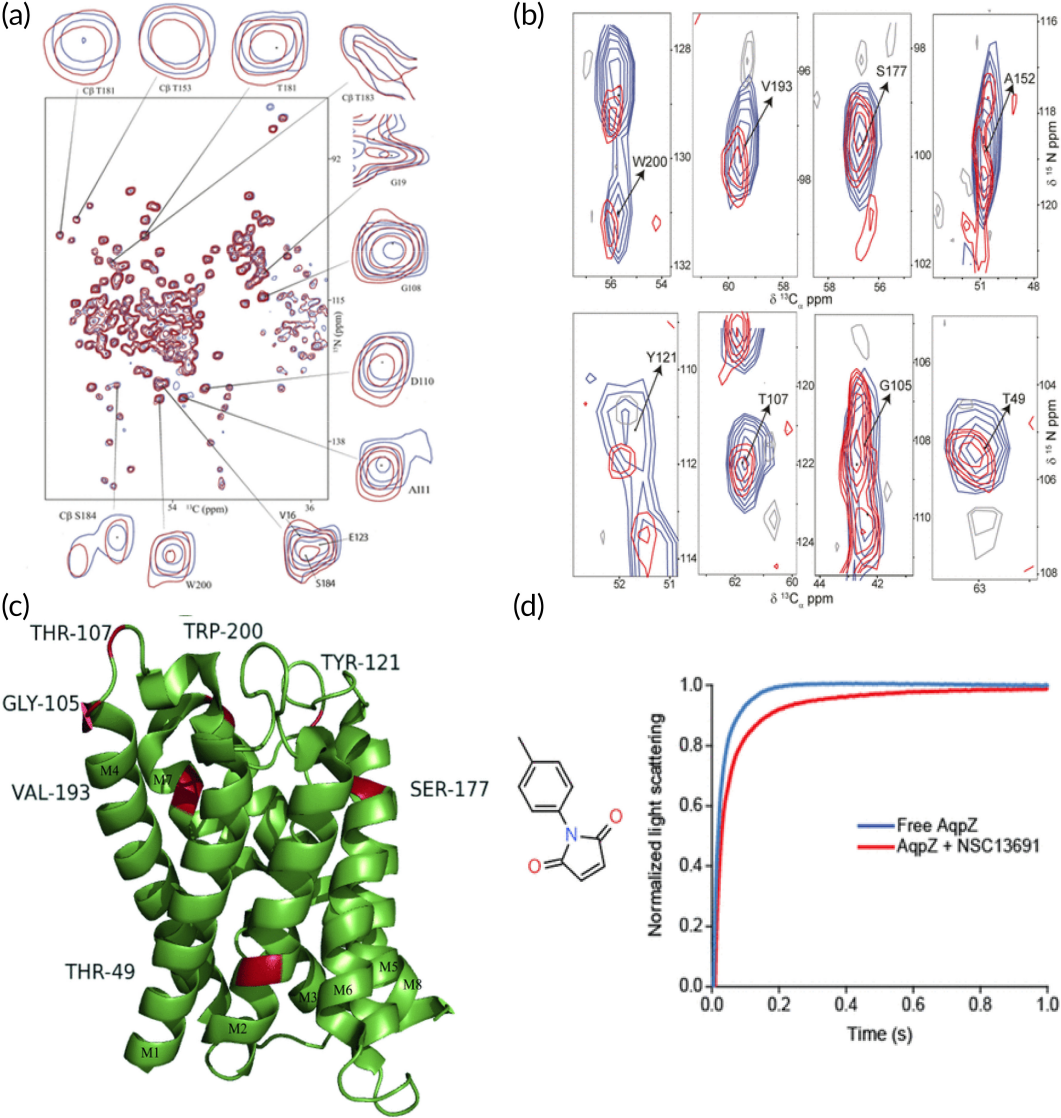
Structure‐based elucidation of inhibitor binding site in AqpZ proteoliposome by solid‐state MAS NMR. (a) Two dimensional (2D)^15^N–^13^C_α_ spectra of AqpZ with inhibitor (NSC13691) (red) and without inhibitor (blue) recorded by solid‐state MAS NMR illustrating that AqpZ–drug interaction leads to perturbation in chemical shift of AqpZ structure with significant perturbations being highlighted. (b) Contour plots of 2D planes (^15^N, 13C_α_) corresponding to AqpZ with inhibitor (red) and without inhibitor (blue) extracted from 3D NCACX spectra with the assigned peaks indicating significant chemical shift perturbations of more than 0.2 ppm in both 13Cα and 15 N planes. (c) Mapping of the ApZ–drug interaction site onto the crystal structure (1RC2). The residues that have undergone significant chemical shift perturbations have been highlighted in red. (d) The inhibitor NSC13691 blocks AqpZ function as characterized by a stopped‐flow water permeability assay in AqpZ proteoliposomes. A reduced water permeability of AqpZ is observed as characterized by a significant decrease in the rate constant of the AqpZ proteoliposomes shrinkage. *Source*: Figure 9a–d is reproduced with permission from reference 34, Copyright 2018, Springer Nature

Once lead compounds are identified, high‐resolution studies are required to understand the binding sites of small molecule modulators as well as how they perturb the MP of interest. An example of a high‐resolution study is using solid‐state MAS NMR to investigate the binding and structural change of a small molecule water channel inhibitor (NSC13691) to AQPZ (Figure 9a–c) in proteoliposomes, together with their functional inhibition in a stopped‐flow water permeability assay (Figure 9d).^34^ Future directions in HTS for drug discovery can include mass production of nano‐vesicles^209^ and construction of cell‐like structures such as membrane‐based nanoreactors, artificial cells,^210^ or synthetic cell chassis, and the exploitation of microfluidic devices to increase the throughput in MP studies and drug discovery.^211^

## 8 SUMMARY AND FUTURE PERSPECTIVES

In this review, we provided extensive insights into MP studies using proteopolymersomes, specifically in the types of proteins studied, techniques used to study MP structures and functions and complications involved in these studies. Multiple factors affecting MP studies in proteopolymersomes suggest that the silver‐lining in engineering proteopolymersomes that provide the most relevant structural and functional information lies in detailed optimization and controllability of the processes for amphiphilic polymers self‐assembly, protein synthesis, and reconstitution. The advancement to incorporate enzymes, chaperone proteins, and DNA to these synthetic membranes to generate artificial cells^212^, ^213^ or nanoreactors^214^, ^215^, ^216^ will further propel promising platforms not only to study MPs but also investigate a wide range of cell functions and processes that initiate from the formation of proteopolymersomes.

**FIGURE 10:**
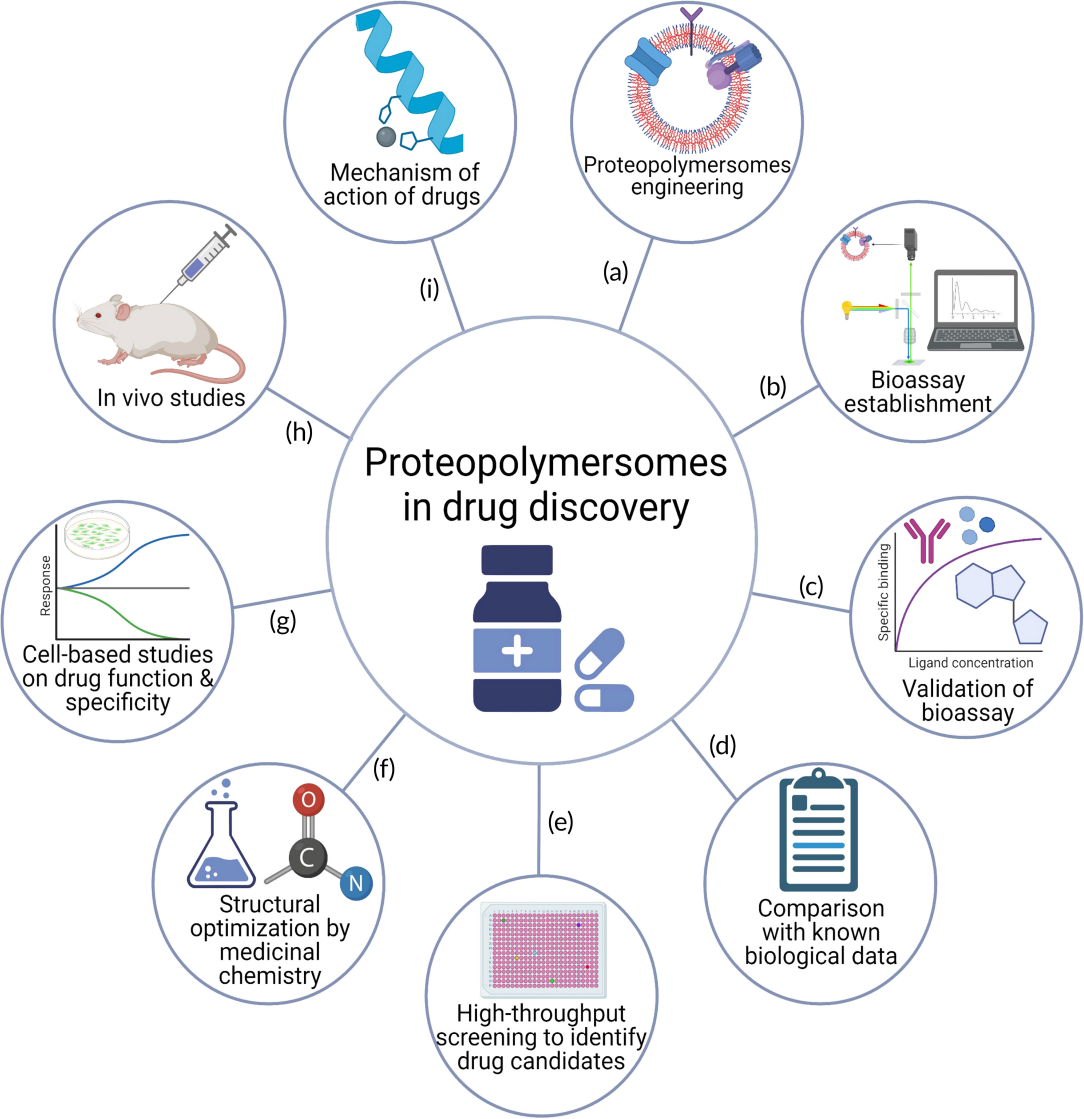
Prospective research directions and a roadmap for using proteopolymersomes in drug discovery. (a) Proteopolymersomes engineering using suitable block copolymers and gene/protein or interest. (b) Bioassay establishment with proteopolymersomes using appropriate detection methods such as FRET measurement for structural change or fluorescent leakage assay for channel functional assay. (c) Validation of bioassay using positive controls that are known to alter protein structure or modulate protein function in proteopolymersomes. (d) Compare the structural and functional data obtained from proteopolymersomes with known biological data from literature to benchmark the performance of the bioassay established with proteopolymersomes to decide whether to proceed with high‐throughput screening (HTS) or more optimizations may be required. (e) HTS of suitable chemical libraries using proteopolymersomes to identify hit compounds or potential drug candidates for further testing. (f) Potential drug candidates may undergo structural optimization by medicinal chemistry to obtain analogs with improved pharmacological properties, which will be tested in proteopolymersomes for their functions. (g) Cell‐based studies will be conducted to determine the efficacy of the potential drug candidates in modulating cellular functions as well as elucidate their specificity to the protein target. (h) Use of animal models such as mice to validate the efficacy of potential drug candidates in vivo. (i) To elucidate the mechanism of action of the potential drug candidates using proteopolymersomes, including their binding sites, how they perturb the protein of interest as well as whether they act through competitive or allosteric mechanisms. Schematics were created with BioRender.com.

We further detailed the feasibility and current implementation of reconstituted MPs in synthetic membranes, including proteopolymersomes and MP bound polymeric bilayer, to be used in HTS for therapeutic discovery. Although multiple examples in using proteo‐nanovesicles to study the modulation of MP structures and functions by pharmaceutical compounds have been reported, there are limited studies using proteo‐nanovesicles, in particular proteopolymersomes, as HTS platforms in drug discovery. The main obstacles in using proteopolymersomes in the HTS process lie in the lack of robust and scalable production methods to produce large batches of proteopolymersomes with perfect monodispersity and reproducibility.^217^, ^218^ Once these obstacles are overcome, we propose that proteopolymersomes are suitable to be used as HTS platforms to discover novel therapeutics targeting the reconstituted protein of interest and provide corresponding recommendations as well as a roadmap for using proteopolymersomes in drug discovery pipeline (Figure 10).

1. Development of proteopolymersome‐based screening platform: Exploiting the use of polymersomes in drug discovery can potentially be adapted to all membrane‐bound protein targets through careful selection of suitable reconstitution methods and polymer mixtures to engineer the desired proteopolymersome as a screening platform (Figure 10a). This platform, in principle, allows the incorporation of any MP for which the complementary DNA is available. An initial characterization of the engineered proteopolymersomes should be performed to ensure good quality control. To achieve inserted MPs of better quality, chaperones or enzymes may be added to assist the MP folding.^110^ Furthermore, other cellular components such as organized metabolic reactions and gene expression mechanisms may be included in distinct spatial compartments in the proteopolymersomes^18^, ^218^ to achieve the engineering of synthetic cells as a screening platform, which would be compatible in their biological functionalities to drug screening using living cells. This is followed by the characterization of the structure and function of the reconstituted MP to validate its physiological folding and functions. The proteopolymersome constructed will need to be coupled to a detection method such as fluorescence or luminescence measurements to establish the screen platform with sensing capability (Figure 10b). The adaptation into high‐throughput formats, including accurate dispensing of proteopolymersomes into multiwells, calibrations, and determination of limit of detection should be optimized.
2. Validation of the screening platform and benchmarking against known biological data: The proteopolymersomes based screening platform can be tested with positive controls known to modulate the MP structure (e.g., conformational dynamics) and function (e.g., ligand binding) to ensure that the MPs are responding to all protein‐specific stimuli and to determine the compound efficacy or binding affinities to the MPs (Figure 10c). Testing with positive controls will also determine the assay quality Z‐factor and coefficient of variation of the screening platform.^219^ Negative controls that are known to target other MPs should also be tested to ensure that they do not perturb the target MPs of interest and there is high specificity of compounds to the reconstituted proteopolymersomes. Mutations in MPs that might change their signals as well as other proteins such as native ligands can be used to validate the MP functions in proteopolymersomes. These parameters can be benchmarked with known biological data or results obtained from other cell‐based assays that have been previously conducted to assess the performance of the screening platform (Figure 10d). If the experimental values fall outside the acceptable range of the known data, the engineering of proteopolymersomes must be further optimized to obtain data that corroborate with other studies to ensure accuracy and consistency.
3. Automation‐assisted HTS: The established screening platform with high mechanical stability can be used in combination with automations such as multiwell plate‐readers, microfluidics or SPR to assist drug screening using proteopolymersomes, which will facilitate high‐throughput and rapid turnover in identifying MP modulators (Figure 10e). New technologies enabling HTS and the scaling up of the production of proteopolymersomes for drug screening can be developed to facilitate the hit identification process. Hit compounds can undergo further structural optimization by medicinal chemistry to improve on the pharmacological properties of the potential drug candidates (Figure 10f). These compound analogs should be tested with proteopolymersomes to ensure that they are still targeting the protein of interest. Furthermore, the binding sites and the chemical groups of the analogs that interact with the MPs can be elucidated by structural studies using proteopolymersomes. This will allow chemists to make better informed decisions in designing the structures of the analogs for improved binding and potency.
4. Assessment of the efficacy of hit compounds in cell‐based assays and animal models: The efficacy of the hit compounds should be determined using secondary or orthogonal assays typically through cell‐based studies (Figure 10g) as well as in appropriate animal models (Figure 10h). Both the functionality and specificity of the hit compounds should be determined at this stage. Typically, the hit compounds are specific if it does not have an effect in cells and animal models containing the knockout of the MPs of interest. The use of proteopolymersomes in drug discovery has an added advantage of having higher specificity than conventional cell‐based biosensors containing other biological components. From these studies, the pharmacokinetics, pharmacodynamics, and drug biodistribution should also be determined.
5. Elucidation of the mechanism of action of the lead drug candidate using proteopolymersomes: The protein–drug interaction sites and whether the drug acts through a competitive or allosteric mechanism can be elucidated using proteopolymersomes (Figure 10i). Proteopolymersomes containing mutations in the protein of interest can be utilized to confirm the binding sites where a lack of key binding residues will reduce drug binding. The binding sites of the lead drug candidates can be determined by obtaining high‐resolution crystal structure of drug bound proteins by cryo‐EM or x‐ray crystallography. Structural perturbation or conformational change of protein of interest induced by the drug candidates can be resolved by NMR spectroscopy. Furthermore, protein complexes or coupling systems in proteopolymersomes would enable the investigation of the effect of drugs not only on the target MPs but also on other downstream proteins in the pathways.

## AUTHOR CONTRIBUTIONS

**Chih Hung Lo:** Conceptualization (lead); funding acquisition (equal); writing – original draft (equal); writing – review and editing (equal). **Jialiu Zeng:** Funding acquisition (equal); writing – original draft (equal); writing – review and editing (equal).

## CONFLICT OF INTERESTS

There are no conflicts of interest to declare.

## Data Availability

Data sharing is not applicable to this article as no new data were created or analyzed in this study.
